# The Inheritance Pattern of 24 nt siRNA Clusters in Arabidopsis Hybrids Is Influenced by Proximity to Transposable Elements

**DOI:** 10.1371/journal.pone.0047043

**Published:** 2012-10-31

**Authors:** Ying Li, Kranthi Varala, Stephen P. Moose, Matthew E. Hudson

**Affiliations:** 1 Department of Crop Sciences, University of Illinois at Urbana-Champaign, Urbana, Illinois, United States of America; 2 Energy Biosciences Institute, University of Illinois at Urbana-Champaign, Urbana, Illinois, United States of America; University of Leeds, United Kingdom

## Abstract

Hybrids often display increased size and growth, and thus are widely cultivated in agriculture and horticulture. Recent discoveries demonstrating the important regulatory roles of small RNAs have greatly improved our understanding of many basic biological questions, and could illuminate the molecular basis for the enhanced growth and size of hybrid plants. We profiled small RNAs by deep sequencing to characterize the inheritance patterns of small RNA levels in reciprocal hybrids of two *Arabidopsis thaliana* accessions, Columbia and Landsberg *erecta*. We find 24-nt siRNAs predominate among those small RNAs that are differentially expressed between the parents. Following hybridization, the transposable element (TE)-derived siRNAs are often inherited in an additive manner, whereas siRNAs associated with protein-coding genes are often down-regulated in hybrids to the levels observed for the parent with lower relative siRNA levels. Among the protein-coding genes that exhibit this pattern, genes that function in pathogen defense, abiotic stress tolerance, and secondary metabolism are significantly enriched. Small RNA clusters from protein-coding genes where a TE is present within one kilobase show a different predominant inheritance pattern (additive) from those that do not (low-parent dominance). Thus, down-regulation in the form of low-parent dominance is likely the default pattern of inheritance for genic siRNA, and a different inheritance mechanism for TE siRNA is suggested.

## Introduction

Hybrid plants have wide utility in agriculture, yet studies of gene expression variation are more commonly conducted using inbred model organisms. Heterosis (also known as hybrid vigor) refers to the phenomenon where a hybrid, produced by crossing two genetically diverse parents of the same or different species, displays enhanced growth compared to the parents. Desirable traits for hybrid crops include greater biomass, fertility or tolerance to abiotic/biotic stress. Hybrids have therefore been utilized for centuries to increase crop yield. Scientific interest in hybrids and heterosis also has a long history, dating back to Darwin [1]. Three popular genetic models have been proposed to explain the phenomenon of heterosis in hybrids, “dominance” (where detrimental recessive alleles from one parent are complemented by dominant beneficial alleles from the other parent in the hybrid); “pseudo-dominance” (where two or more recessive detrimental alleles exist in tightly linked loci in repulsion phase) and “over-dominance” (where the interaction of different alleles at a single locus leads to phenotypic differences in the hybrid). Results from prior studies have supported and contradicted these three models to varying degrees [2]–[5]. Since these interpretations are based on classical genetic theory, it is not yet clear how epigenetic phenomena and small RNA fit into these models, and whether knowledge of small RNA inheritance could lead to improved mechanistic understanding and prediction of heterosis [6].

Each of the above models have implications for how gene expression may behave in a hybrid relative to the parents. Thus, global RNA expression profiling has become a popular approach to study hybrid biology. This type of experiment can reveal what genes are differentially expressed, how they are regulated and potentially shed light on whether a given gene or gene network contributes to the phenotypes altered by hybridization. Genome-scale gene expression assays have been performed with a wide range of plant tissues in various developmental stages, and different proportions of non-additive and additive gene expression patterns have been reported [7]–[14]. Although the specific conclusions differ between individual studies, most agree that gene expression levels in hybrids are largely within the parental range. Most studies report that the majority of genes show additive expression patterns, and non-additive inheritance is also usually observed at a lower frequency [7], [8], [12]–[14]. Considered collectively and similar to the interpretations from genetic mapping studies, many modes of inheritance of gene expression patterns appear to be relevant to heterosis [7], [9], [11].

The analysis of inheritance patterns for gene expression in hybrids also offers insights into modes of regulatory variation. Allele-specific expression assays reveal the relative contributions of cis- and trans-acting regulation [9], [15]–[17]. A study of global gene expression in rice hybrids investigated the prevalence of insertions/deletions in the promoters of differentially expressed genes in the hybrids, and proposed a cis-regulation model [18]. Conversely, expression QTL analysis in maize hybrids suggested that the majority of differentially expressed genes in hybrids were paternally controlled in a trans-manner [19]. Additionally, it was also shown that epigenetic regulation might contribute to heterosis [20]. Overall, the regulation of gene expression after hybridization is complex, and the wide range of interpretations from gene expression profiling experiments following hybridization likely reflects the different tissues, genotypes and species employed. Evidence for a number of different models has been found and our current understanding of the genetic basis of heterosis remains insufficient to modify or optimize its phenotypic effects.

The recent discovery of small RNA has added another component to transcriptomic analysis [21], [22]. Small RNAs are short, regulatory non-coding RNAs with a size of 20–30 nt [23]. MicroRNA (miRNA) and small interfering RNA (siRNA) are two major types of small RNA in plants [23]. MicroRNAs are typically 20–22-nt small RNAs produced from miRNA genes encoding transcripts with imperfect fold-back secondary structure [24]. miRNAs target specific mRNAs by sequence pairing for directed transcriptional cleavage or translational repression. Known miRNAs are largely involved in regulating plant development [23]. siRNA are 21–24-nt small RNAs generated from endogenous sources such as transposable elements and tandem repeats, or exogenous sources like RNA viruses and transgenes. The great majority of endogenous siRNAs are 24-nt heterochromatic siRNAs, whose major role is to repress transposon activity by methylation to maintain genome integrity [23]. Two other types of siRNA, cis-nat siRNA (produced from overlapping protein-coding genes) and tasi-RNA (triggered by 22-nt miRNA) have also been described [25]–[27] with certain duplex structures as well as some 22 nt miRNAs having the capacity to trigger secondary siRNA [28].

Small RNA has been proposed as a possible mediator of the previously reported trans-regulation in hybrid gene expression [19]. He et al. [20] also suggested a possible role for the 24-nt siRNA on heterochromatin silencing in heterosis. The influence of hybridization on small RNA profiles has been the subject of a number of recent studies [6], [20], [29]–[31]. Although the changes observed in the different experiments suggest complex regulatory variation at individual small RNA producing loci, when considered at a genomic scale a common theme has emerged. Unlike previous microarray experiments examining mRNA expression, these studies consistently find non-additive expression of small RNA that predominantly follows a pattern of low-parent inheritance. Certain classes of genes, especially those involved in the circadian clock, DNA methylation and photosynthesis [32], [33], have been implicated as likely mediators of differential phenotypes of hybrids; it is possible if not likely that small RNA is the source of differential regulation of these genes in hybrids. Accordingly, changes in mRNA expression and DNA methylation were also shown to correlate with non-additive small RNA expression. Thus, an emerging biological consensus is that the distinct phenotypes seen in hybrids may be influenced by small RNA via DNA methylation causing non-additive changes in the expression of mRNA.

In this study, we compared the global small RNA profiles of *Arabidopsis thaliana* (L.) Heynh accessions Columbia (Col) and Landsberg *erecta* (Ler) with their reciprocal hybrids. Unlike prior studies that sampled mature organs containing a complex mixture of cell types and were typically not replicated, we analyzed replicated samples of meristematic tissues where the enhanced growth of hybrids is most likely programmed. Arabidopsis is widely used as a model organism for the study of hybrid biology [34]. The genome of the Col accession is fully sequenced [35], a draft version of the Ler genome is available [36], and ten years of annotation work by the community makes the Arabidopsis genome one of the best-annotated plant genomes. Here, we identify genomic regions that differentially express small RNAs between hybrids and their parents, and correlate this to neighboring genomic features such as transposable elements.

## Results

### Arabidopsis reciprocal hybrids exhibit hybrid vigor

Two Arabidopsis accessions, Ler and Col, were reciprocally crossed to produce the hybrids Col×Ler (Col maternal, abbreviated to CL) and Ler×Col (Col paternal, abbreviated to LC). Concurrently Ler and Col individuals were emasculated and manually selfed to produce inbred parent lines Ler×Ler (LL) and Col×Col (CC) as controls to compare with the hybrids. Arabidopsis, when manually crossed, tends to show increased seed size and plant vigor, most likely as a result of fewer siliques and, in the case of sub-optimal timing or technique, fewer embryos per silique in manually crossed plants. Inbred seeds produced by manual selfing, as opposed to naturally selfed seed, ensures that our observations of transcriptional and phenotypic differences between the inbreds and hybrids are not due to artifacts of manual crossing.

**Figure 1:**
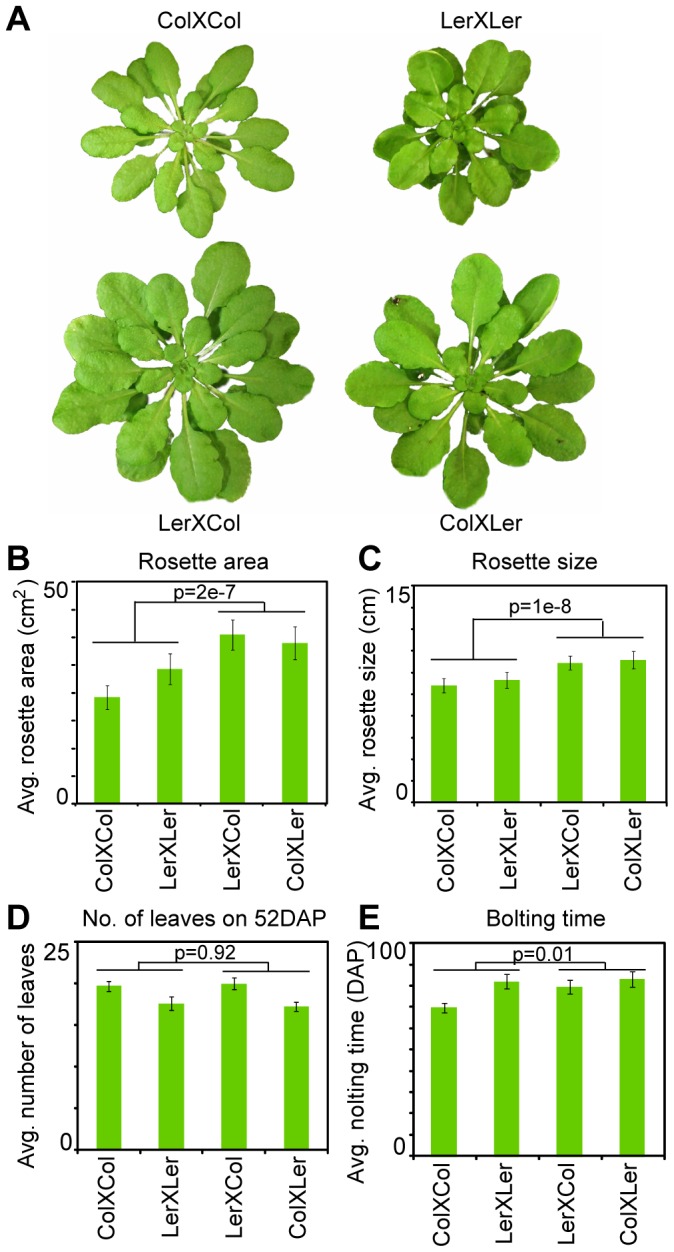
Heterotic phenotypes. A. Example picture showing one plant of each of the four genotypes at 52 days after planting (DAP). B. Rosette area of the inbreds and hybrids measured as the green leafy area from horizontal plane photographs of plants at 52DAP. C. Rosette sizes of the inbred and hybrid plants, measured as the sum of the lengths of the longest leaf and second longest leaf on 52DAP. D. Number of leaves of the hybrids and inbreds on 52DAP (leaf length>0.5 cM). E. Bolting time of the four genotypes was measured. In all panels, 30–40 individual plants of each of the four genotypes (inbred parents LerXLer, ColXCol and hybrids LerXCol, ColXLer) were measured (except for bolting time where data from 5–7 plants are shown). Error bars represent the 95% confidence interval.

Both hybrids as well as their inbred parents were grown at 20°C in short days. To assess the level of heterosis, we compared rosette area, rosette size, leaf number and bolting time of the inbreds and hybrids. As anticipated based on previous work [37], above-high-parent heterosis was observed in both rosette area and rosette size in Arabidopsis hybrids at 52 days after planting (DAP) (Figure 1, A–C). In contrast, the number of leaves at 52DAP or the bolting time did not show high-parent heterosis (Figure 1, D&E). The hybrids in this study thus exhibited heterosis phenotypes in rosette size and rosette area to a slightly greater degree than previously described [37]. This difference may be a result in differences in culture practices, or could be a result of the plants used in this study being grown under short day conditions. These phenotypes likely result from accumulation of more biomass during development, rather than faster development in the hybrids compared to the inbreds, because two indicators of developmental stage, leaf number and bolting time, do not show changes in the hybrids.

### Overview of the small RNA profiles in hybrids and inbreds

Both hybrids as well as their inbred parents were grown under short days until they reached the 20-leaf stage (54DAP to 56DAP), when plants were harvested in liquid nitrogen for small RNA profiling. The genotypes of all plants used for small RNA profiling were confirmed using the SSR marker *nga106* [38]. We specifically investigated the global small RNA profiles of the apical region of hybrids and inbreds (Supplemental Fig. S1). As the shoot apical meristem is programmed to develop into reproductive organs, we hoped to capture important genetic events, potentially leading to the high fertility of hybrids, by comparing the small RNA profiles of the rosette shoot apex, including the meristem, in hybrids and inbreds before the transition from vegetative growth to reproductive growth. Since the apical region is small, the plants were grown under short day conditions to allow it to reach a larger size to maximize the amount of available small RNA, and also RNA was pooled from multiple plants. Four biological replicates of the hybrids (LC and CL) and three biological replicates of the inbreds (LL and CC), each consisting of an equimolar pool of four single-plant-derived total RNA samples, were used for small RNA library construction and Illumina sequencing. Two technical replicates were performed for each biological replicate. Such replication of the small RNA sequencing assay allows sufficient statistical power to reliably identify differentially expressed small RNAs.

**Figure 2:**
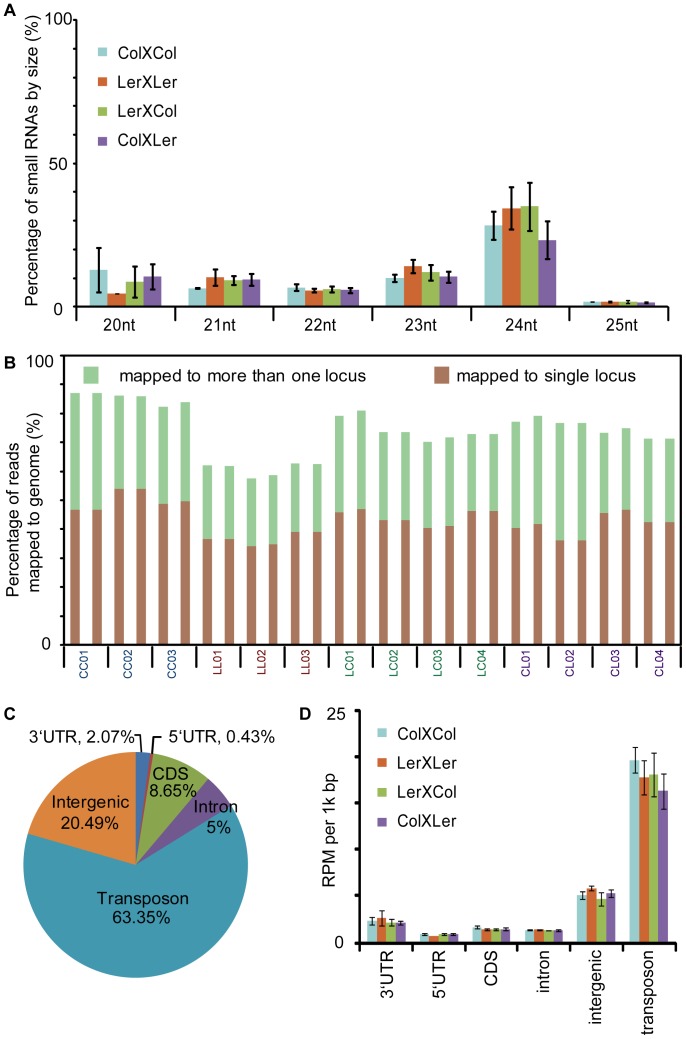
Size distribution and genome mapping of the small RNAs. (A) Percentage of small RNA reads from each genotype by size. (B) Percentages of small RNA reads (abundance) mapped to the reference genome. CC: ColXCol; LL: LerXLer; CL: ColXLer; LC: LerXCol. The numbers on the X-axis differentiate the biological replicates. Two technical replicates were included for each biological replicate. (C) Percentage of small RNA reads mapped to different genomic components. The mean percentage across all libraries was plotted. (D) Average abundance of small RNA mapped to individual genomic features. The abundance of small RNA mapped to each genomic feature was first normalized to the total genome-mapped reads in each library and then to the length of the genomic feature (reads per kilobase per million, RPKM). The error bars represent the standard error of the mean.

A total of 67 million raw reads were generated using the Illumina GAIIx for this experiment. Raw reads, after adaptor trimming and preprocessing, were first mapped separately for each library to Arabidopsis rDNA and tRNA using the alignment software novoalign (http://www.novocraft.com/). On average 30.49% of the total reads mapped to tRNA and rDNA, which is comparable with the rRNA and tRNA content previously reported in other actively dividing Arabidopsis tissues [39]. Because we considered small RNAs from tRNA and rRNA loci were likely to be derived from degradation rather than small RNA biogenesis pathways, reads mapping to tRNA and rDNA were removed from further analysis. After removing rDNA- and tRNA-related small RNA, 24-nt small RNA is the most abundant small RNA species among all libraries (Figure 2A). A one-way ANOVA test showed that there was no significant difference in the small RNA size distribution among the four genotypes.

Remaining reads from each library were mapped separately to the Arabidopsis Columbia genome (TAIR9, http://arabidopsis.org/) again using novoalign with only perfect matches allowed. On average, 85% of the reads from CC (Columbia) libraries mapped perfectly to the TAIR9 (Columbia) genome, while 61% of the reads from LL (Landsberg *erecta*) libraries mapped perfectly to the TAIR9 genome (Figure 2B). A lower number of aligning LL reads is expected considering the known sequence variation between Ler and Col. Allowing up to two mismatches increased the percentage of mapped reads by 7% for CC libraries and 22% for LL libraries, but also lead to an increase in ambiguous mapping since many more reads mapped to two or more sites in the genome. Therefore, only perfect matches were used for further analysis. The average percentage of mapped reads in the hybrids (LC and CC) was 75%, approximately the average between the Col and Ler parent values (Figure 2B). Considering all libraries, 58% of the mapped reads uniquely aligned to one genomic locus in the reference genome (Figure 2B).

While miRNA is generally considered to target protein-coding genes, siRNA can potentially regulate the activity of various genomic components. Based on the TAIR9 annotation, we determined that 63.35% of small RNA reads mapped to transposons, 20.49% of reads mapped to intergenic regions, and 16.16% mapped to genic regions (protein coding genes, not including miRNA genes). The reads mapping to genic regions can be further divided into those matching CDS(8.65%), introns (5%), 5′-UTRs (2.07%) and 3′-UTRs (0.43%) (Figure 2C). To study the production of small RNA on a per-kilobase basis, total reads of small RNA mapped to a given type of genomic feature were first normalized to the total genome-mapped reads for each library, then normalized to the size (bp) of the genomic feature type in the Arabidopsis genome (reads per kilobase per million (RPKM)). Small RNAs associated with transposon sequences were more frequent (averaging 17 RPKM), followed by intergenic regions, then genes (Figure 2D). Within genes, small RNAs showed the highest mapping frequency to the 3′ UTR, probably because miRNAs preferentially target 3′ UTRs (Figure 2D).

### Positional cluster-assisted differential analysis of small RNA level

siRNAs that are related to each other in function or in biogenesis are likely to be found densely clustered on both strands of a siRNA-producing genomic locus (e.g. a transposon or a targeted gene) [39] (Supplemental Fig. S2A). Also, any given miRNA/miRNA* pair should be located at two adjacent positions of the miRNA gene in the transcribed strand, forming a “sparse cluster” [39] (Supplemental Fig. S2B). It is therefore useful to cluster small RNAs with a proximity-based algorithm [39]. In our study, if a small RNA maps to genomic position X, while another small RNA maps to genomic position Y, and the distance between X and Y is less than 500 bp, a “cluster” starting at X and ending at Y is created. The cluster building continues until the next neighboring small RNA read maps greater than 500 bp away – a new cluster is then initiated for the next read on the chromosome. 500 bp was chosen as the threshold because it generated a reasonable average cluster size and a manageable total number of clusters, and it was used in previous studies [39]. Small RNA reads from all four genotypes were pooled together to build a total of 56,654 clusters across the five Arabidopsis chromosomes (Supplemental Fig. S3A), with cluster sizes ranging from 16 bp to 366,826 bp (Supplemental Fig. S3B). The mean size of the clusters was 854 bp, while the median was 181 bp. The small RNA expression level of each cluster is the sum of abundance of all small RNA reads mapping to the cluster, normalized to the total mapped reads from the respective library (giving a figure in reads per million (RPM) per cluster, hereafter referred to as cluster count). A mapping-loci-assisted weighting method was used to prevent over-counting of small RNAs caused by small RNA mapping to multiple genomic loci. Only clusters with greater than 5 RPM in at least one library were included for further differential analysis (represented by blue dots in Supplemental Fig. S3B).

To identify clusters that are differentially expressed (DE) between hybrids and inbreds, a one-way ANOVA was performed (false discovery rate (FDR) controlled at 5% [40]) with the null hypothesis that Col, Ler and the hybrids have similar cluster counts. The ANOVA step hence identified clusters differentially expressed to a statistically significant degree either between the two parents or between parents and hybrids. To study hybrid small RNA inheritance pattern as well as parental effect, we did a total of three ANOVA analyses. In set I, differentially expressed small RNA clusters among LL, CC and both hybrids (CL and LC pooled together) were identified. In set II, small RNA clusters that are significantly differentially expressed among LL, CC and LC were calculated. In set III, differentially expressed small RNA clusters among LL, CC and CL were calculated. Overall, 361 clusters were identified in set I, 106 DE clusters were identified in set II, and 133 DE clusters were identified in set III (Table 1). The larger number of DE clusters identified in Set I is likely a result of increased statistical power gained by treating the two hybrids as a larger pool of replicates. While a substantial number of clusters were identified that were differentially expressed, no clusters were identified in the hybrids that were not expressed in one or the other parent.

**Table 1:** Numbers of differentially expressed (DE) clusters with different small RNA expression patterns.

	both hybrids	LerXCol	ColXLer	both hybrids	LerXCol	ColXLer
	*counts*			*%*		
DE^a^ clusters	361	106	133	-	-	-
Additive	152	39	50	36.8%	37.6%	42.1%
Non additive	209	67	83	63.2%	62.4%	57.9%
LP^b^	163	52	60	49.1%	45.1%	45.2%
HP^c^	5	0	4	0.0%	3.0%	1.4%
AHP^d^	0	1	4	0.9%	3.0%	0.0%
BLP^e^	2	1	1	0.9%	0.8%	0.6%
between LP and mid-parent	39	13	14	12.3%	10.5%	10.8%
between HP and mid-parent	0	0	0	0.0%	0.0%	0.0%

a Differentially expressed.

b Low parent like.

c High parent like.

d Above high parent.

e Below low parent.

**Figure 3:**
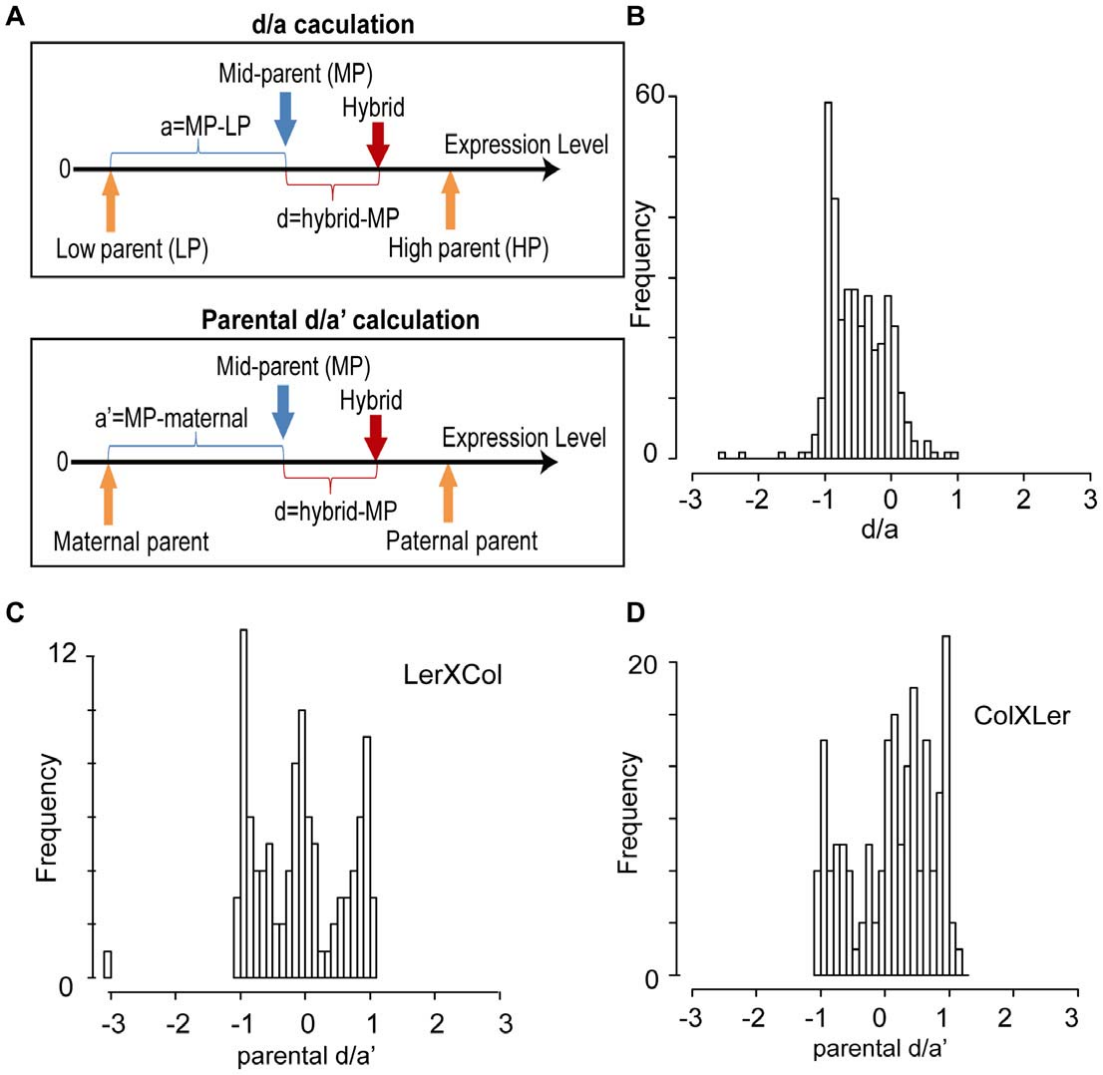
d/a plots showing the dominant/additive hybrid inheritance patterns relative to parents. (A) Diagram demonstrating the calculation of d/a value and parental d/a′ value. d/a = 1 indicates the hybrid small RNA level is similar to the high parent while d/a = −1 indicates the hybrid small RNA level is similar to the low parent. d/a = 0 means the hybrid small RNA level is the same as the mid-parent value. In parental d/a′ plots, d/a′ = 1 indicates hybrid small RNA level is similar to the male parent while d/a′ = −1 indicates the hybrid small RNA level is similar to the female parent. d/a′ = 0 means the hybrid small RNAlevel is the same as the mid-parent value. (B) d/a plot of the DE clusters in set I (analysis considering both reciprocal hybrids as a single dataset). (C) Parental d/a′ plot of the DE clusters identified in set II (analysis considering the hybrid with the Landsberg line as female only). (D) parental d/a′ plot of the DE clusters identified in set III (analysis considering the hybrid with the Columbia line as female only).

To compare the small RNA level of hybrids to their inbred parents, the ratios of dominant value to additive value (d/a values) [11] were calculated for all DE clusters identified from the three ANOVA analyses (Figure 3; Supplemental Figure S4A&B). The d/a histogram of DE clusters identified in set I, where both hybrids were considered, suggested that the great majority of clusters showed a d/a value between 0 and -1, with the highest peak around -1 (Figure 3B). Such a distribution indicates that the small RNA levels of the hybrids are on average lower than the mid-parent value (d/a = 0), with the majority appearing as a low parent like pattern and mid-parent/additive pattern. The d/a histograms of set II and set III, where either Ler×Col or Col×Ler alone was considered as the hybrid in the ANOVA study, showed very similar patterns (Supplemental Fig. S4). To investigate possible paternal/maternal effects, parental d/a′ values were also calculated for set II and set III (Figure 3A, C&D). We cannot exclude a minor maternal effect in Ler×Col and a minor paternal effect in Col×Ler, but the size of the effect, if present, is sufficiently small to be of questionable significance.

### Hybrid small RNA inheritance patterns

**Figure 4:**
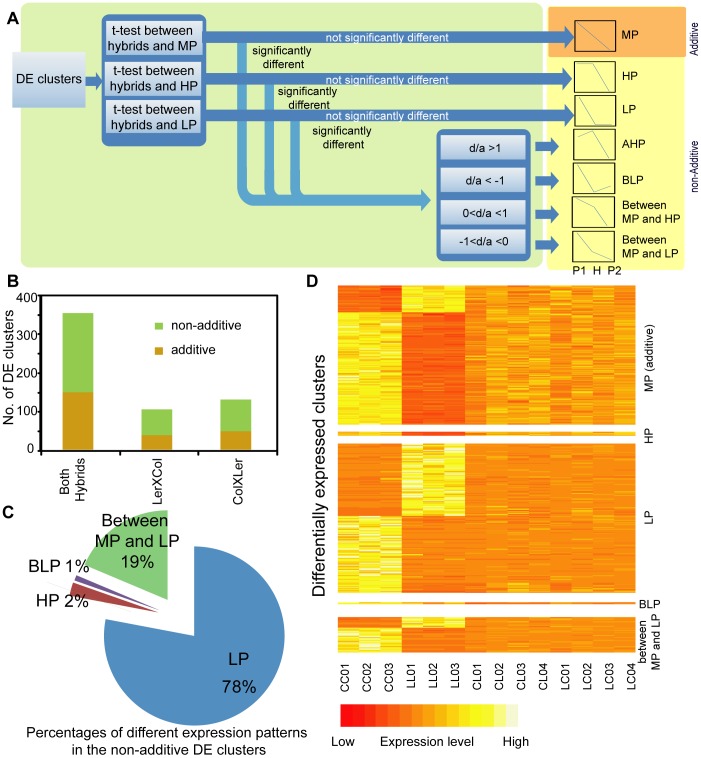
Approach and results of hybrid inheritance pattern fitting. (A) Diagram showing how a differentially expressed small RNA cluster (DE cluster) was categorized as one of the seven patterns: Mid-parent like (Additive), high parent like (HP), low parent like (LP), above high parent (AHP), below low parent (BLP), between mid-parent and HP, and between mid-parent and LP. Significance cutoff for t-tests was 0.05. (B) Numbers of additive and non-additive DE clusters identified when (i) both LerXCol and ColXLer were combined as the hybrid group (ii) only considering LerXCol as the hybrid and (iii) only considering ColXLer as the hybrid. (C) Percentages of different patterns in the non-additive DE clusters identified in set I (analysis considering both reciprocal hybrids). (D) Small RNA levels of DE clusters identified in set I (analysis considering both reciprocal hybrids), grouped by their hybrid inheritance pattern. Each row represents the normalized expression level (cluster count) of a DE cluster. The columns represent individual biological replicates of the four genotypes. MP: mid-parent; HP: high parent; LP: low parent; BLP: below low parent.

The production level of any small RNA clusters of the hybrid was classified into one of the following seven patterns, depending on its quantitative relationship with the production level of the parents: mid-parent like (MP), high parent like (HP), low parent like (LP), above high parent (AHP), below low parent (BLP), between LP and MP, and between HP and MP (Figure 4A). MP is also known as the additive pattern, while the other patterns are collectively called non-additive patterns. All DE clusters in our study were assigned to one of the seven patterns, by means of t-test and d/a value as previously described [11] (Figure 4A). Overall, slightly more DE clusters fell into the non-additive category (58% to 63%) than the additive category (37% to 42%) (Figure 4B; Table 1). Among the non-additive DE clusters, a substantial majority (72% to 78%) displayed a LP like level (Figure 4C; Supplemental Fig. S5A&B), followed by a level between MP and LP (17%–19%). Among the seven inheritance patterns, the biggest portion showed an LP pattern (45% to 49%), followed by MP (37% to 42%) and between LP and MP (11% to 12%) (Figure 4D; Supplemental Fig. S5, C&D; Table 1). Very few clusters showed HP and BLP patterns (Figure 4D; Supplemental Fig. S5, C&D; Table 1). The low number of BLP patterns could be partly attributed to the very low levels of many LP values (109 of 163 clusters had RPM<5), making it difficult for hybrid values in those 109 clusters to fall significantly below LP. Therefore, the majority of the non-additively expressed small RNA clusters displayed a reduction in small RNA production level compared to the MP value. Finally, almost all the clusters (96%–99%) showed a hybrid level within the parental range. Altogether, low-parent dominance was the most prevalent single inheritance pattern, as seen also in the d/a plot (Figure 3; Supplemental Figure S4, A&B).

The DE clusters determined by the three analyses share a large overlap (Supplemental Fig. S6). Among the 75 DE clusters shared by the three sets, 64 of them have the same pattern in all three sets (36 are LP, 23 are additive, 5 are between LP and MP). Eleven clusters that were categorized into different patterns in different analyses alternate between LP and “between LP and MP” (8 clusters), or between MP and “between LP and MP” (2 clusters), or between MP and LP (1 cluster). Overall, the three sets of analysis agreed strongly that 1) the small RNA production clusters is repressed in hybrids overall and that 2) the predominant patterns are MP and LP.

### Functional annotation of the differentially expressed clusters

**Figure 5:**
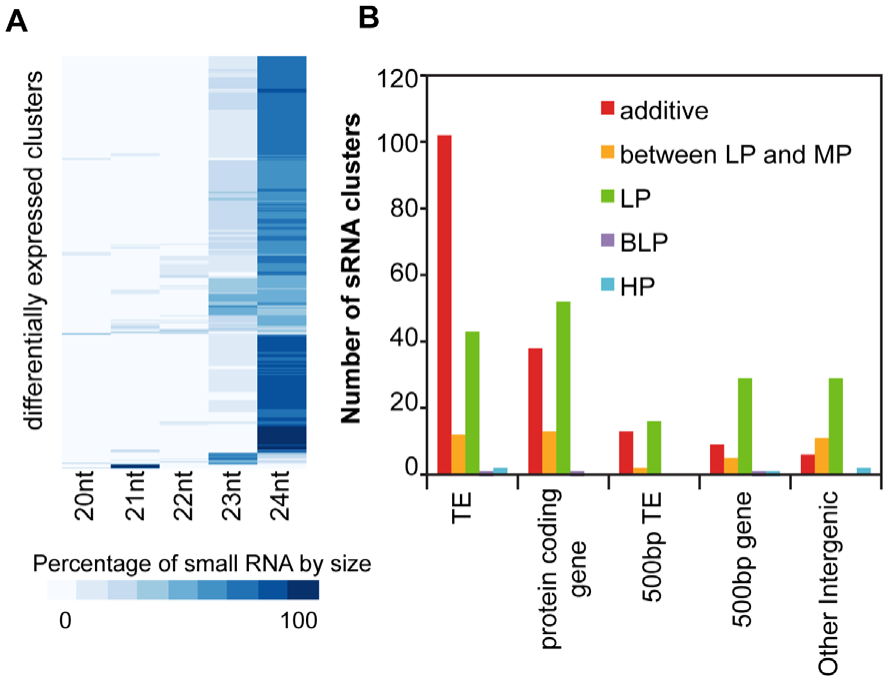
Annotation of differentially expressed small RNA clusters. (A) Size distribution of small RNAs in the differentially expressed clusters and (B) relationship between the genomic origins and the hybrid inheritance patterns of the differentially expressed clusters identified in set I (analysis considering both reciprocal hybrids). 500 bp gene: within 500 bp upstream or downstream of a protein coding gene; 500 bp TE: within 500 bp upstream or downstream of a transposable element; LP: low parent; HP: high parent; MP: mid-parent (additive); BLP: below low parent.

To investigate the biological relevance of the low parent dominance pattern in small RNA level, we studied the likely role of the DE clusters by analyzing the size and sequence similarity of the small RNA as well as the secondary structure and genomic annotation of the clusters. First we studied the size distribution of the small RNA associated with differentially expressed genomic clusters. For the great majority of the DE clusters (353/361) identified in set I, the 23-nt/24-nt class was the most abundant (Figure 5A), while the 20/21/22-nt class was the most abundant in only 8 clusters (Figure 5A). The size composition of the DE clusters strongly suggests that most of these DE clusters are more likely to be associated with 24-nt siRNA rather than miRNA. To further test this, a few steps were taken to search for any possible miRNA genes within the DE clusters. Firstly, the highest expressed small RNA from each DE cluster was compared with the known mature miRNA database (miBase.org) using SSEARCH [41] (p-value cutoff: 0.001), which did not find any significant hits. Next, we checked whether the genomic sequence around the highest expressed small RNA in each DE cluster can be folded into a stable fold-back structure using UNAfold (mfold.rna.albany.edu). Only 10 clusters out of 361 were found to possess favorable fold-back structures. However, in 5 of these clusters, small RNAs were mapped to transposons, therefore the fold-back structures could be attributed to inverted repeats present in TEs. In another three clusters, small RNAs were distributed across a protein-coding gene, thus are unlikely to have been processed as miRNAs. In the remaining two clusters, the fold-back structures were formed by pairing between simple tandem repeats; thus they do not completely satisfy the required structural features expected of a miRNA precursor. Finally, we visualized relative small RNA abundance using Gbrowse2 (http://gmod.org) and examined all the DE clusters to look for clusters with features similar to potential miRNA generating loci (Supplemental Fig. S2B). However, the vast majority of the DE clusters showed features characteristic of loci that generate siRNAs (Supplemental Fig. S2A). Overall, we conclude that in the meristematic tissue sampled in this study, the great majority if not all of the small RNA clusters that are differentially expressed in hybrids compared to the inbreds produce 24-nt siRNAs.

Using the GFF annotation (TAIR9) of the Arabidopsis genome, we were able to query whether the small RNA clusters corresponded to an annotated transposable element (TE), a protein-coding gene or an intergenic region. In 160 out of the 361 DE clusters, the majority of the small RNAs from the clusters mapped to TEs. In 104 clusters, the majority of the small RNAs mapped to protein-coding genes. The functional descriptions of those genes are listed in Supplemental Table S1. This left 97 clusters where small RNAs do not directly map to either a transposon or a gene. For these clusters, the closest TE/gene within 500 bp, if any, was then determined. Among these 97 clusters, 31 have a transposable element as the closest annotated element, and 45 have a protein-coding gene as the closest annotated element.

When the genomic annotation of the small RNA clusters was associated with the hybrid inheritance pattern, we observed that clusters associated with TEs typically exhibit additive inheritance. In contrast, small RNAs associated with protein-coding genes are largely repressed in the hybrids relative to the mid-parent value. Thus the predominant LP-dominance observed for sRNA clusters is more characteristic of protein-coding genes than transposable elements (Figure 5B). This trend is also observed for DE clusters identified from analysis sets II and III (Supplemental Fig. S7). Compared to a total of 368 DE clusters, the TE-associated clusters had a significantly (P<0.001) greater than expected number of additively inherited pattern (102 of 160). The proportion of the protein-coding genes that were additively inherited (38 of 104) or LP inherited (52 of 104) was not significantly different from the distribution of the total set of DE clusters. In order to further explore whether this result was robust to genotypes or the tissue sampled, we directly compared our results to those reported by Groszman et al. 2011 [6] (Supplemental Figure S8). Small RNA clusters differentially expressed between the parents, where the hybrid shows a mid-parent expression value in the Groszmann et al. study, frequently show a difference between the mid-parent value and the hybrid level in our study (Supplemental Fig. S8 A&B). This discrepancy between the two studies could be expected given the different genotypes and tissues involved, as well as the differences in statistical power. However, the d/a values (Supplemental Fig. S8C) (and to some extend the ratio of hybrid to midparent values (Supplemental Fig. S8D)) are remarkably consistent between this study and the earlier study for clusters where hybrids show an expression value below mid-parent levels.

**Figure 6:**
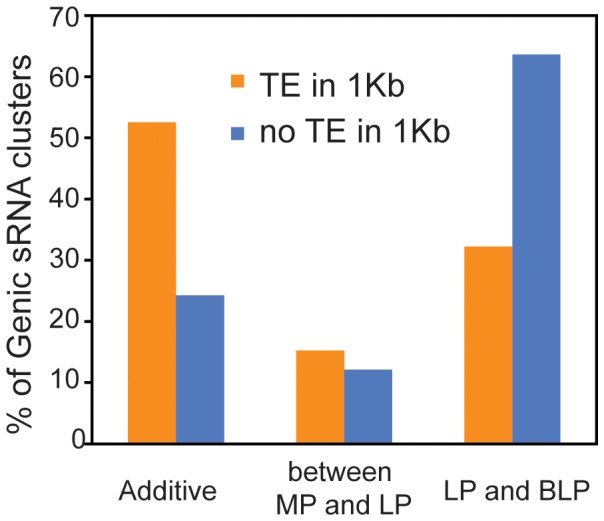
Effect of neighboring transposons on the inheritance patterns of genic siRNA clusters. The percentage of clusters of siRNA from protein-coding genes classified into mid-parent (additive), between mid parent and low parent, and low parent and below low parent (LP and BLP) where a transposable element (TE) was present within 1 kilobase of the gene is shown.

We observed a consistent association between inheritance pattern and whether or not a cluster is close to a transposon; however, many protein-coding genes show additive (transposon-like) inheritance. Methylated TEs close to genes have been associated with deleterious effects on those genes and purifying selection [42], [43]. We thus investigated whether genic clusters of siRNA within 1 kb of a TE showed a different inheritance pattern to those that are not. We found that 64% of genic siRNA clusters without a TE within 1 kb showed LP or BLP patterns, and 24% showed additive expression (Figure 6). The presence of a TE within 1 kb profoundly alters this pattern, with only 32% of genic siRNA clusters with such a neighboring TE being LP or BLP, and 53% being additively inherited. This difference from the expected pattern is significant at the P<0.05 level.

**Figure 7:**
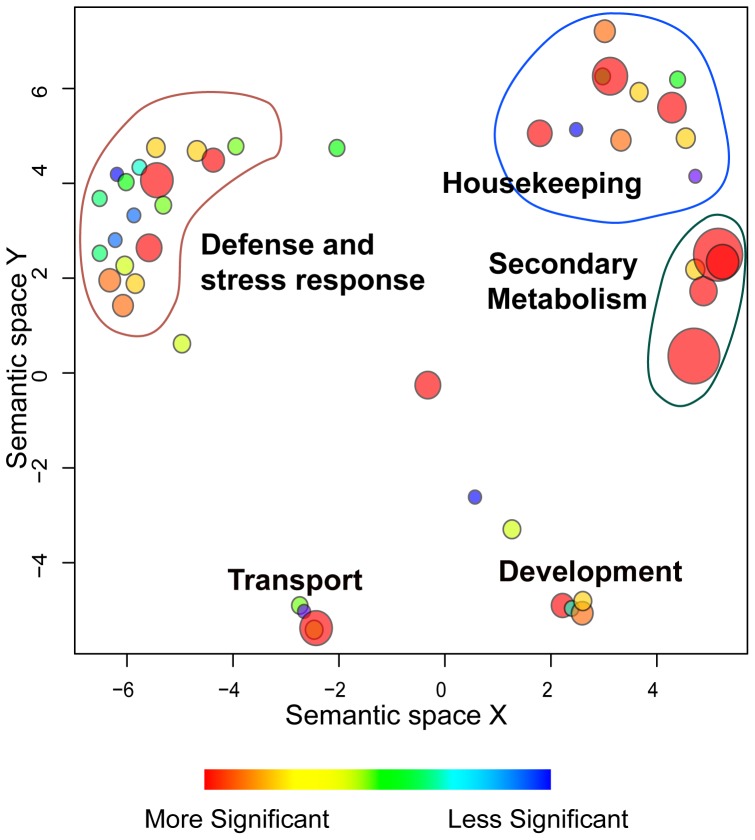
Gene Ontology (GO) terms identified in the genes associated with low-parent like small RNA level and between LP and MP small RNA level. The GO terms are represented by circles, visualized in a semantic similarity-based scatter plot, where similar GO terms are close to each other, using software ReviGO. The area of the circle is proportional to the significance of the over-representation of the GO term (-log10 p-value). The color of the circles represents the statistical significance of the over-representation of the GO term, as shown in the legend. The GO term annotations are in Supplemental Table S2.

While additive inheritance of small RNA in the hybrid implies that the regulation of the two parental alleles of the locus is not altered, the clusters displaying low-parent dominance (or between LP and MP patterns) are loci whose expression regulation is altered upon hybridization. Thus, since this group is enriched with sequences matching protein-coding genes, regulation of these genes by small RNA may be altered in the hybrids. A Gene Ontology (GO) term list was prepared for the group of genes that are associated with LP-like or between-MP-and-LP small RNA level. Statistical significance of the enrichment of the GO terms with respect to the Arabidopsis whole gene set was determined using a p-value calculated with the hypergeometric distribution (Supplemental Table S2). The GO terms and their associated P-values were then visualized with ReviGO [44]. Genes with predicted functions in secondary metabolism and defense and stress response genes are over-represented in the non-additively inherited siRNA clusters (Figure 7; Supplemental Table S2).

## Discussion

Recent advances in short-read sequencing technology, especially the improvement in single-lane yield and multiplexing techniques, allow sufficient replication and depth of sampling to detect with statistical confidence specific genomic regions showing differential expression of small RNAs in hybrids compared to their parents. Among all the small RNA-generating loci with significant differential expression between hybrids and inbreds, approximately 58% were non-additively inherited. To our initial surprise, we found that a low-parent like small RNA level in the hybrids is the predominant non-additive pattern, followed by the “between low-parent and mid-parent” pattern; three recent publications [6], [20], [30] also reported similar results. Therefore, the most significant feature of the non-additive small RNA expression in the hybrids is down-regulation of small RNA production in the F_1_. That this is consistent among experiments despite differences in species, tissue type, developmental stage, and experimental design, suggests that the down regulation of small RNA loci in hybrids may likely be a universal phenomenon.

We found that most of the differentially expressed small-RNA-generating loci produce 24-nt small RNA, therefore they are unlikely to be miRNA loci. Indeed, no differentially expressed miRNA were observed, whether the miRNAs are defined by similarity to existing miRNAs or by secondary structure prediction. We therefore concluded that the differentially-expressed small-RNA loci between Ler and Col are all likely to be siRNA-generating loci. A similar result was reported in another Arabidopsis hybrid [6]. We believe this is consistent with the finding that miRNAs most often functional to regulate highly conserved developmental processes, which continue to operate in hybrids that are morphologically similar to their parents, differing primarily only in relative size. In contrast, siRNAs are more commonly involved in short-term evolutionary processes such as gene silencing. However, this conclusion could be limited by the tissue type and developmental stage examined in this study, because the proportion of 21-nt to 24-nt small RNA could vary depending on the tissue type and plant developmental stage in question.

We then investigated which subtypes of siRNA were differentially regulated in our inbred/hybrid system. A few different types of siRNA have been reported. A large portion of small RNAs are heterochromatic 24-nt siRNAs, which guide DNA methylation and histone methylation machineries to homologous loci for transcriptional silencing of transposons or genes [23], [45]. Another type of siRNA, trans-acting siRNA, are 21-nt siRNA generated by 22-nt miRNA-directed cleavage of TAS gene transcripts [26]. The cis-nat siRNA [25] are produced from overlapping, tail-to-tail transcription units, which are 21-nt or 24-nt in length, and work in *cis* or on closely related genes [25], [46]. In our study, most differentially expressed clusters turned out to be consistent with 24-nt siRNA generating loci. Among them, 44% featured siRNA generated from transposable elements and 29% featured siRNA mapped to protein coding genes. In a smaller portion (27%) of the differentially expressed clusters, the small RNAs mapped to intergenic regions. The transposon-mapped 24-nt siRNA are most likely heterochromatic 24-nt siRNA, whose role is to guide the methylation of transposable elements to maintain genome integrity. The interpretation of biogenesis and function of the genic 24-nt siRNA is not very clear. They are unlikely to be cis-nat siRNA because detailed investigation did not reveal genomic structures where portions of adjacent genes overlap in inverse orientations. It is possible they are generated via cryptic promoters that produce antisense RNA from a single gene locus without the need for a cis-nat pair. These siRNAs are likely associated with directing the formation of heterochromatin, where whole genes, gene fragments or pseudogenes are included in a heterochromatic region and are hence less accessible to transcription.

We also found that the transposon 24-nt siRNA were mostly inherited in an additive manner, while the genic siRNAs were largely down regulated to approximately the low parent level in the hybrids. A predominantly low-parent inheritance of protein-coding genic siRNA has been reported in other Arabidopsis hybrids [6], [30] although rice hybrids [20] did not appear to show the same clear result, nor did Arabidopsis tetraploids [29]. Our finding that TEs show a different pattern implies that a different mechanism of inheritance may operate for these elements than for protein-coding genes. We also, intriguingly, found that the substantial number of protein-coding genes showing additive expression tended to have nearby TEs, while those showing low-parent type expression did not. This finding adds weight to the hypothesis of Hollister and Gaut [42] that silencing of TEs may come with an additional cost by causing deleterious silencing of nearby genes. Interestingly, a very recent study [47] has shown that TE-associated, differentially methylated genomic regions are accompanied by siRNAs that are upregulated in response to biotic stress, which are also coupled to mRNA expression of the transposon and/or proximal gene.. While the expression of such TE-linked genic siRNA is predominantly additive, it is possible that the combination of reduction of such siRNA to mid-parent levels combined with the low-parent inheritance of other genic siRNAs could reduce deleterious silencing and thus contribute to heterosis.

It is generally accepted that 24-nt transposon-associated siRNA has an important role in plant genome maintenance and evolution. In genomes with very abundant repeats and transposons, such as the maize genome, impairment of the heterochromatic siRNA pathway causes severe developmental defects [48]. The additive inheritance of heterochromatic siRNA from the different varieties of TEs combined in hybrids could maintain or increase the effectiveness of TE silencing, leading to a more stable genome. In the case of a genome with active transposons, such as maize, this could substantially reduce the cost to control TE activity in hybrids. However, Barber et al. [31] recently showed that a reduction in the level of 24 nt siRNAs does not lead to a decrease in the extent of heterosis in maize. While Arabidopsis does not have the abundant active TEs of maize, our results indicate that a number of siRNA loci are non-additively inherited in the cross used for this study, perhaps indicating the biology of this hybrid is more complex than the previously reported single-locus heterosis conferring the vigor of the Ler X Col hybrid [49].

A large subset (29%) of 24-nt siRNA generating loci that are down-regulated in hybrids are mapped to protein-coding genes. The majority of the genic siRNA clusters are down-regulated in the hybrids. In these cases, the down-regulation of 24-nt siRNAs may indicate that the expression level of target genes may be altered in the hybrids due to altered epigenetic control. GO term annotation of these genes highlighted secondary metabolism and stress/defense responses as significantly over-represented terms. Timely and sufficient expression of stress and defense response-related mRNAs could play a role in hybrid vigor, since a number of agriculturally important hybrids are known to have superior stress tolerance [50]. While the faster growth of hybrids may also make them more vulnerable to some types of stress, a general feature of stress tolerance in some hybrids is one of agronomic value that is little understood at the molecular level. Our finding that many genic small RNA clusters are predicted to function in stress and defense response could be a clue to how the greater stress tolerance of hybrids may be achieved. The links shown by Dowen et al. [47] between transposon-associated clusters and siRNA that are upregulated by biotic stress make an intriguing combination with our finding that biotic stress genic siRNAs are disproportionately inherited in a non-additive manner. Furthermore, a portion of DE clusters feature 24-nt siRNAs mapped to intergenic regions within 500 bp of a protein coding gene, suggesting that those siRNAs may possibly mediate methylation of genomic regions adjacent to genes (e.g. promoter or enhancer sequences), thus affecting the gene activity. Recently it was reported [30], [51] that regions of altered siRNA are correlated with altered methylation and gene expression in Arabidopsis hybrids. Although the magnitude of changes in mRNA levels was relatively low, they were observed along with non-additive decreases in some types of methylation signatures. This non-additive inheritance of both small RNA and epigenetic markers provides a new phenotype for hybrids, although its importance to programming enhanced growth remains to be investigated.

Finally, the different inheritance patterns of the TE-derived siRNA (additive) compared to siRNAs derived from protein-coding genes (low-parent dominance) suggests that different regulatory mechanisms may control siRNA production from these two types of genomic loci in hybrids. In the Arabidopsis genotypes used here, active transposable elements have not been reported; thus it seems less likely that repression of transposons could lead to increased growth in the hybrid. However, there is evidence that TEs close to genes have a negative influence subject to purifying selection, presumably as a result of deleterious silencing of nearby genes [42]. In the hybrid, genic siRNAs are predominantly inherited in a low-parent pattern, unless there is a neighboring transposon (Figure 6). Thus, a number of deleterious silencing effects of 24 nt siRNA may be reduced in the hybrid, as a result of lower levels of genic siRNA. In maize, the reduced capacity for amplification of 24-nt sRNAs decreases overall vigor, but not the degree of heterosis [31]. Deleterious siRNA-mediated silencing may act to repress gene expression (as previously described [47]) and thus growth. Alleviation of this repression in hybrids suggests a possible mechanism for heterosis.

In summary, the majority of differentially expressed small RNA clusters between Ler and Col parents are non-additively inherited in the F_1_ hybrids. Approximately half of the differentially expressed small RNA clusters overlap TEs, and these predominantly show an additive pattern in the hybrids. Many differentially-expressed siRNA clusters are derived from protein-coding genes, among which stress and defense response genes are over-represented. These predominantly display a non-additive, low-parent-like pattern, unless there is a TE present within 1 kb. The results of this study suggest that differential regulatory mechanisms of siRNA from protein-coding genes and transposable elements in hybrids may be important in understanding the mechanisms of hybrid biology and possibly heterosis.

## Materials and Methods

### Plant material

Arabidopsis accessions Columbia (Col) and Landsberg *erecta* (Ler) were obtained from the Ohio State University Arabidopsis Stock Center (ABRC). These inbred lines were reciprocally crossed to generate hybrids LerXCol (LC, Col as the paternal parent and Ler as the maternal parent) and ColXLer (CL, Ler as the paternal parent and Col as the maternal parent). Col and Ler were also manually emasculated and selfed separately to produce LerXLer (LL) and ColXCol (CC) as inbred parents in the comparisons.

Seeds from all crosses were surface sterilized and planted in a soil mix (sunshine mix∶perlite∶vermiculite = 2∶1∶1). They were subjected to stratification for six days at 4°C in darkness and then allowed to germinate and grow in a growth chamber with 130 uM*s-1*m-2 white light for 7 hours a day at 20°C. The short day conditions allow the extension of vegetative growth and thus the analysis of small RNA from a larger meristem, and reduce the complication of differential flowering time effects between inbreds and hybrids.

### Total RNA extraction and small RNA sequencing

Plants of the four genotypes were harvested when they reached the 20-leaf stage (leaf length>0.5 cm) to make sure plants at the same developmental stage were compared. Tissue was collected over 3 days, from 54 days after planting (DAP) to 56 DAP. The tissue collection was done at the same time of day during these three days to remove the complication of potential circadian controlled gene expression. All the plants were collected at least two weeks before bolting, therefore the plants were most likely in the vegetative stage. Each plant was harvested in liquid nitrogen in two parts: the central meristematic tissue was harvested as a 0.8 cm diameter circular sample centered at the apical shoot meristem, for total RNA extraction and small RNA profiling (Supplemental Fig. S1); the remaining leaves were then harvested for marker-assisted genotyping to confirm the expected hybrid/inbred genotypes; the root was discarded (Supplemental Fig. S1).

Leaves collected for genotype confirmation were subjected to genomic DNA extraction and genotyping with an INDEL marker (nga106). Genotypes of plants used for RNA extraction and small RNA sequencing were confirmed by this method to ensure no inadvertent selfs or crosses were included. Total RNA was extracted from the central meristematic region of each genotype-confirmed plant individually, as described previously [52] except for the removal of the LiCl precipitation step. Equimolar amounts of four individuals were pooled together as one biological replicate. Four such pooled biological replicates for each genotype were submitted for Illumina small RNA sequencing. Each biological replicate was sequenced in two different lanes of an Illumina flow cell as technical replicates. To maximize efficiency and reduce cost, eight samples were barcoded and multiplexed in one sequencing lane.

### Measurement of the heterosis phenotype

The measurement of rosette area, rosette size and leaf number included 32 to 44 individuals at 52DAP for each genotype. To measure the area and size of rosettes, horizontal plane photos of plants were taken with a square paper reference. The number of pixels of the green area of a rosette in the photo was then measured using Photoshop. The number of pixels of the reference square was also measured in the same way, based on which the rosette area can be converted from pixels to cm^2^. The rosette size was measured as the sum of the length of the longest leaf (measured as from the center of the rosette to the furthest edge of the leaf) and the second longest leaf, with ImageJ (http://rsbweb.nih.gov/ij/), normalized to the sum of length of the two diagonal lines of the reference square. Eight plants of each genotype were used to measure the bolting time under the described growth conditions.

### Global analysis of small RNA sequence tags

A multiplexed Illumina sequencing assay with 8 samples per lane generated on average 2.3 million raw reads for each sample. Raw reads were preprocessed with a frequency-based short-read clustering algorithm called FreClu [53] (default parameters), where raw reads were quality filtered, adaptor trimmed, replicated reads merged and sequencing-error corrected. FreClu generated on average approximately 0.4 million distinct reads representing approximately 1.7 million reads per library with a size range of 12–31 nt. An in-house Perl script was used to select reads within the size range 16 bp to 31 bp for further analysis. Two biological replicates, one of LL and one of CC, had more than 50% of reads removed after applying the 16–31 nt size filter. This was due to an abundance of small size reads (12–15 nt) in these two libraries, indicating poor quality probably caused by either poor RNA quality or low library construction efficiency. To ensure that only data from high quality sequencing libraries is included, these two biological replicates were removed from further analysis.

After the above filters were applied, four biological replicates of LC and CL (hybrids) and three biological replicates of LL and CC (inbreds) were retained, each with two technical replicates. Reads that mapped to Arabidopsis rDNA sequences (5S rRNA: NCBI Gene ID 4024964; 18S rRNA gene [54]; 25S and 18S gene spacer [55]; 5.8S, 25S and 18S rRNA 3′ region [56]) and Arabidopsis tRNA (http://gtrnadb.ucsc.edu/Athal/) by novoalign (http://www.novocraft.com/) were removed from further analysis. Residual reads were then mapped to the Arabidopsis Columbia genome (TAIR9, http://arabidopsis.org/) using novoalign allowing only perfect matches (t = 0). Reads were also mapped by novoalign to different genomic components including 5UTR, 3UTR, CDS, intron, intergenic region and transposons in TAIR9 assembly separately to characterize the siRNA activity. Reads that perfectly mapped to the Columbia reference genome from all libraries were then pooled together and clustered with a proximity-based algorithm using an in-house Perl script to generate “clusters”. One cluster represents a genomic region that contains mapping loci of small RNAs that are less than 500 bp apart. The total amounts of small RNAs generated from each cluster was then computed for each library by summing abundance of individual small RNA mapped to this cluster, to represent the small RNA production level of this cluster in the library. In some cases, a small RNA matched to multiple loci in the genome (e.g. mapped to N loci in the genome). A weighted count (i.e. the abundance of the small RNA divided by N) was then used as the measurement of contribution of this small RNA to each cluster that it was mapped to. The small RNA level of a cluster was then normalized to total mapped reads from each library as reads per million (giving the “cluster counts” mentioned in this manuscript) for further analysis. The arithmetic mean of cluster counts of two technical replicates was calculated and used for further analysis.

### Small RNA differential analysis

Scatter plots of normalized cluster count data revealed minimal skewing, and therefore the data was deemed suitable for differential analysis (Supplemental Fig. S9). Clusters with small RNA level lower than 5 RPM in all libraries were removed for further analysis. This excluded 46,051 out of the 56,654 clusters, leaving 10,603 clusters for differential analysis. To identify the clusters differentially expressed between hybrids and parents, and to determine the hybrid small RNA pattern relative to the parents, ANOVA followed by pattern matching using the t-test and d/a value [11] was performed as follows: first, an FDR controlled [40] (FDR cutoff 0.05) ANOVA analysis was performed using R (http://www.r-project.org/) to test which clusters are significantly different between LL, CC and hybrids (CL and/or LC); second, clusters that were significantly different were then assigned to one of the following patterns: high parent-like, low-parent like, mid-parent like (additive), between mid-parent and high-parent, between mid-parent and low-parent, above high parent, and below low parent, based on the results of multiple t-tests (pvalue cutoff: 0.05) and the d/a value (Figure 4A). The d/a values were calculated as follows: a = (high parent−low parent)/2; a′ = (paternal parent-maternal parent)/2; d = hybrid-midparent; d/a value = d/a; parental d/a′ value = d/a′ (Figure 3A).

### Cluster annotation

A number of parallel approaches were taken to annotate the differentially expressed (DE) clusters: (i) an in-house Perl script was written to count the small RNAs with different sizes in a cluster. The small RNA size distribution of a cluster is informative in differentiating siRNA-, ta-siRNA- and miRNA-generating clusters. (ii) The highest expressed small RNA from each cluster was used to search against the miRBase mature miRNA database [57] using SSEARCH [41], to identify clusters producing known miRNA. (iii) A short and strand-specific genomic sequence (170 bp) around the highest-expressed small RNA species was tested for folding into a typical hairpin structure (i.e. a likely miRNA precursor) using UNAfold (Version 3.6, http://mfold.rna.albany.edu/) with the following criteria: dG is smaller than -40; over 75% of the bases in the small RNA need to be paired and the length of the complementary sequence (predicted miRNA*) should not be more than 1.5 times the length of the small RNA; finally, no bases in the small RNA or within 10 bases of its end can be in the complementary strand of the stem-loop i.e., the distance between an miRNA and its complement should be at least 20 bases. (iv) The small RNA level data generated in this experiment was visualized in Gbrowse2.0 (http://gmod.org/) using TAIR9 GFF annotation (http://arabidopsis.org/). All DE clusters were visually checked using Gbrowse 2.0 to determine whether small RNAs are from both strands or a single strand, whether small RNA within the clusters mapped mostly to transposons or to genes, and whether the locus is a likely case for cis-nat siRNA generation with siRNA generated from the overlapping part of two tail-to-tail transcripts. The annotation of siRNA-generating genes was obtained from TAIR10 gene descriptions (tair.org). The statistical significance of over-represented inheritance patterns for differently annotated clusters was evaluated using both the hypergeometric distribution and the chi-squared test. In all cases, both tests showed significance at the level described in the Results section.

## Supporting Information

Supplemental Figure S1 **Tissue used in this study.** Arabidopsis plants at the 20-leaf stage were harvested and cryofrozen in liquid nitrogen. The central rosette apex (indicated by the black circle) was collected for total RNA extraction and small RNA sequencing, while the leaves were collected for genomic DNA extraction and genotype confirmation by PCR.
(TIF)

Supplemental Figure S2 **Two example genomic regions to show a typical siRNA cluster and a typical miRNA cluster.** The example siRNA generating cluster (A) contains small RNAs generated from both strands across the cluster, likely associated with the transponson. The example miRNA generating cluster (B) contains only two major species of small RNAs, miRNA and miRNA*. Both small RNAs mapped to the same strand i.e. the transcribed strand of the miRNA gene locus.
(TIF)

Supplemental Figure S3 **Number, size and cluster count of small RNA clusters.** (A) Number of clusters on each chromosome. (B) The average cluster counts (in reads per million, RPM) across libraries plotted against the cluster size. The blue dots represent the clusters with more than 5RPM counts in at least one library i.e. the clusters used for the differential analysis. The green dots represent the clusters that failed the 5RPM cutoff hence were removed from further analysis.
(TIF)

Supplemental Figure S4 **d/a plots of set II (A) (analysis considering the hybrid with Ler as female only) and set III (B) (analysis considering the hybrid with Col as female only).** d/a = 1 indicates the hybrid small RNA level is similar to the high parent while d/a = −1 indicates the hybrid small RNA level is similar to the low parent. d/a = 0 means the hybrid small RNA level is similar to the mid-parent.
(TIF)

Supplemental Figure S5 **Hybrid small RNA inheritance patterns of set II and set III.** (A) Percentages of different small RNA inheritance patterns in the non-additive differentially expressed clusters (DE clusters) identified in set II (analysis considering the hybrid with Ler as female only). (B) Percentages of different patterns in the non-additive DE clusters identified in set III (analysis considering the hybrid with Col as female only). Small RNA levels of DE clusters identified in set II (C) and set III (D) were grouped by their hybrid inheritance pattern. Each row represents the normalized small RNA level (cluster count) of a DE cluster. The columns represent biological replicates. MP: mid-parent; HP: high parent; LP: low parent; AHP: above high parent; BLP: below low parent.
(TIF)

Supplemental Figure S6 **Venn diagram showing the overlapping and unique differentially expressed clusters identified by the three analysis sets.** Set I: combining both LerXCol and ColXLer as the hybrid group; Set II: only considering LerXCol (LC) as the hybrid; Set III: only considering ColXLer (CL) as the hybrid.
(TIF)

Supplemental Figure S7 **Annotation of the differentially expressed clusters (DE clusters) in set II (analysis considering the hybrid with Ler as female only) and set III (analysis considering the hybrid with Col as female only).** (A&C): Size distribution of small RNAs in the DE clusters identified in set II (A) and set III (C). (B&D): Relationship between the genomic origins and the hybrid inheritance patterns of the DE clusters identified in set II (B) and set III (D). 500 bp gene: within 500 bp upstream or downstream of a protein coding gene; 500 bp TE: within 500 bp upstream or downstream of a transposable element; LP: low parent; HP: high parent; MP: mid-parent (additive); BLP: below low parent; AHP: above high parent.
(TIFF)

Supplemental Figure S8 **Comparison of differentially expressed sRNA clusters identified in this replicated study compared to unreplicated dataset reported by Groszmann et al., 2011** [**6**] **.** The d/a value (A&C) and log2 of hybrid to midparent ratio (B&D) are plotted from our study (X axis) versus the Groszmann et al, 2011 study (Y axis) for both mid-parent like sRNA clusters (A&B) and lower than mid-parent sRNA clusters (C&D). H = hybrid; HP = high parent; LP = low parent; MP = midparent.
(TIFF)

Supplemental Figure S9 **Scatter plots showing small RNA levels of all clusters in the six binary combinations of four genotypes.** In each plot, the base 2 logarithm of the mean of reads per million (RPM) value for a given cluster across biological replicates is shown. The red line represents a straight line with slope = 1.
(TIF)

Table S1 **Genes identified in the differentially expressed clusters.**
(DOC)

Table S2 **GO terms identified among the genes associated with down-regulated small RNAs.** Plot X and plot Y are the coordinators of the GO terms in the semantic space, generated by REVIGO. The p-value was calculated by hypergeometric distribution with the Arabidopsis whole gene set as background.
(DOC)
