# Supplementary figures and images for: The Inheritance Pattern of 24 nt siRNA Clusters in Arabidopsis Hybrids Is Influenced by Proximity to Transposable Elements
Source: PLoS One. 2012 Oct 31;7(10):e47043. doi: 10.1371/journal.pone.0047043 (PMC3485269; doi:10.1371/journal.pone.0047043)

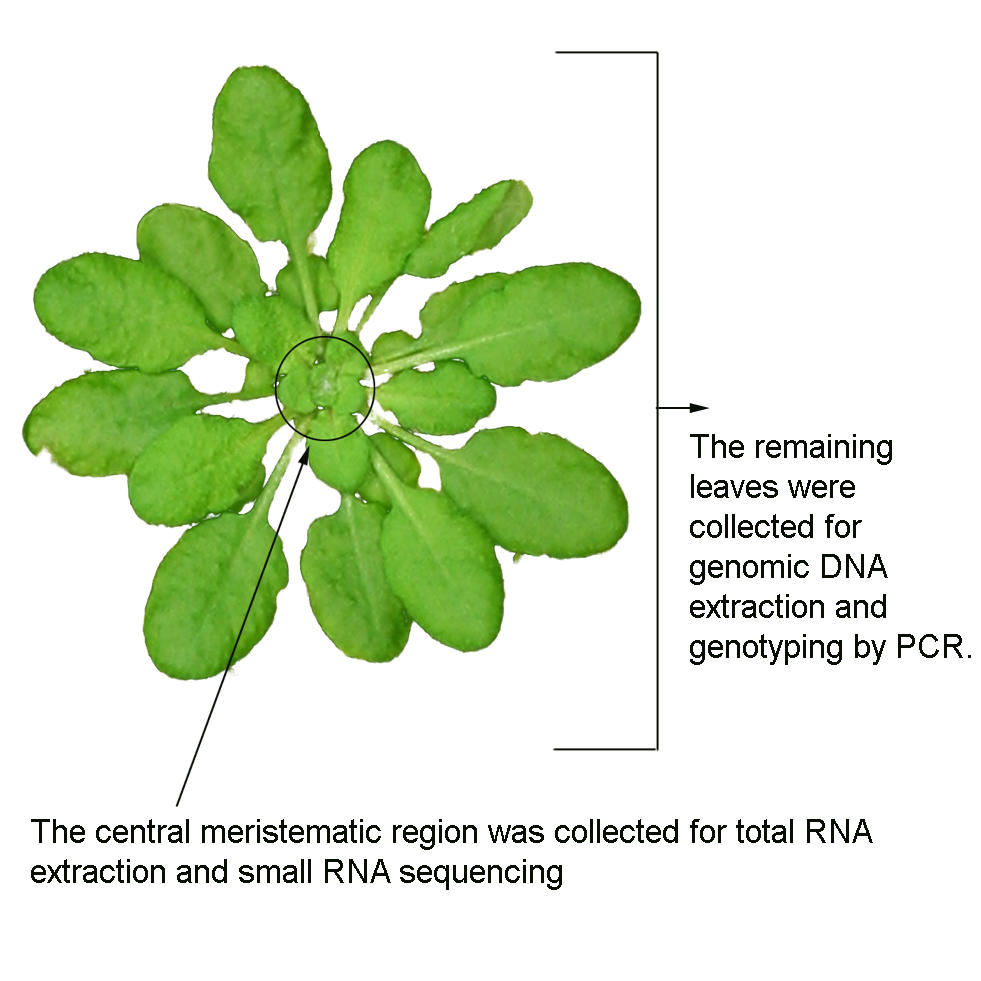

Supplement: Supplemental Figure S1 — Tissue used in this study. Arabidopsis plants at the 20-leaf stage were harvested and cryofrozen in liquid nitrogen. The central rosette apex (indicated by the black circle) was collected for total RNA extraction and small RNA sequencing, while the leaves were collected for genomic DNA extraction and genotype confirmation by PCR. (TIF) [file pone.0047043.s001.tif]

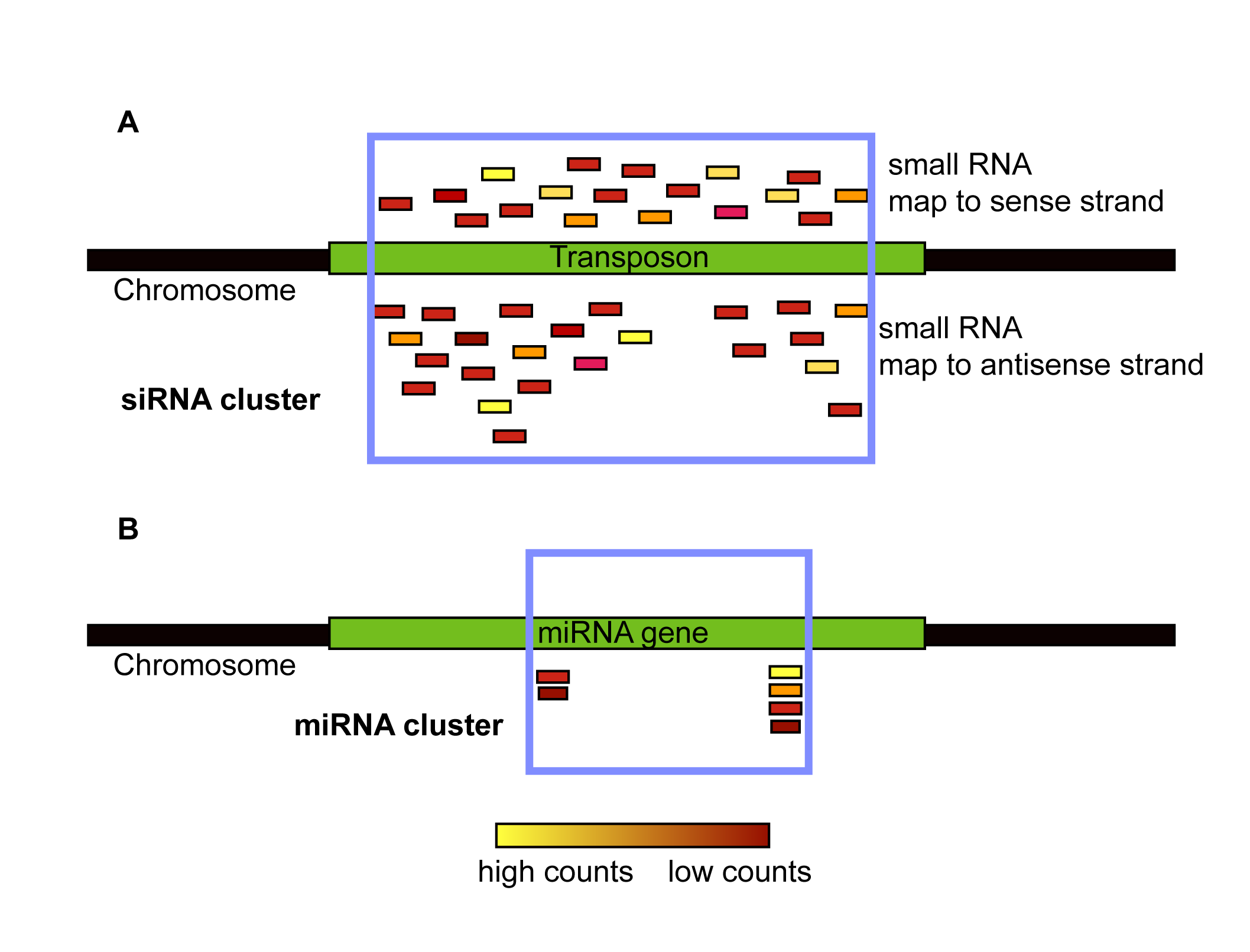

Supplement: Supplemental Figure S2 — Two example genomic regions to show a typical siRNA cluster and a typical miRNA cluster. The example siRNA generating cluster (A) contains small RNAs generated from both strands across the cluster, likely associated with the transponson. The example miRNA generating cluster (B) contains only two major species of small RNAs, miRNA and miRNA*. Both small RNAs mapped to the same strand i.e. the transcribed strand of the miRNA gene locus. (TIF) [file pone.0047043.s002.tif]

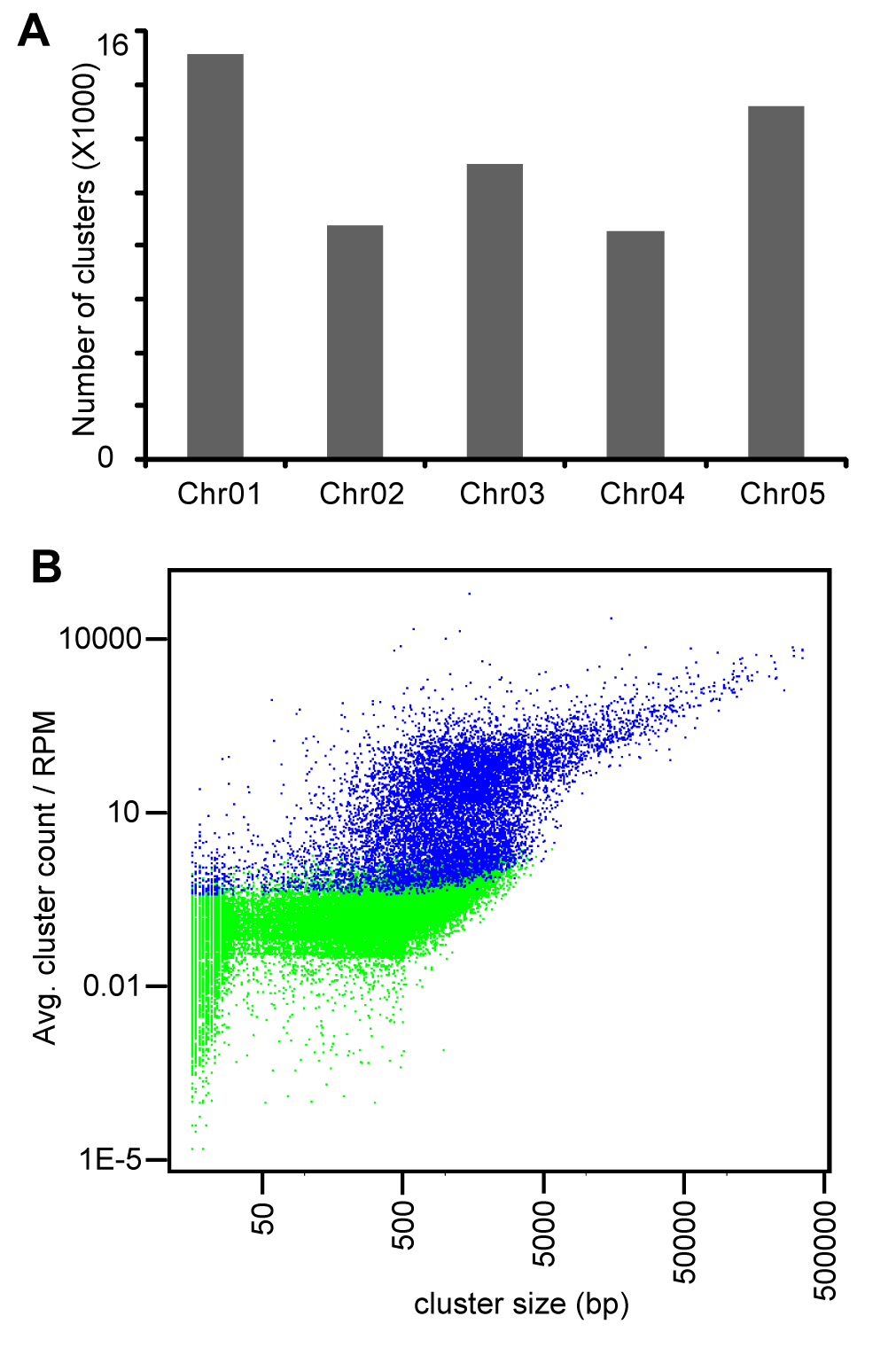

Supplement: Supplemental Figure S3 — Number, size and cluster count of small RNA clusters. (A) Number of clusters on each chromosome. (B) The average cluster counts (in reads per million, RPM) across libraries plotted against the cluster size. The blue dots represent the clusters with more than 5RPM counts in at least one library i.e. the clusters used for the differential analysis. The green dots represent the clusters that failed the 5RPM cutoff hence were removed from further analysis. (TIF) [file pone.0047043.s003.tif]

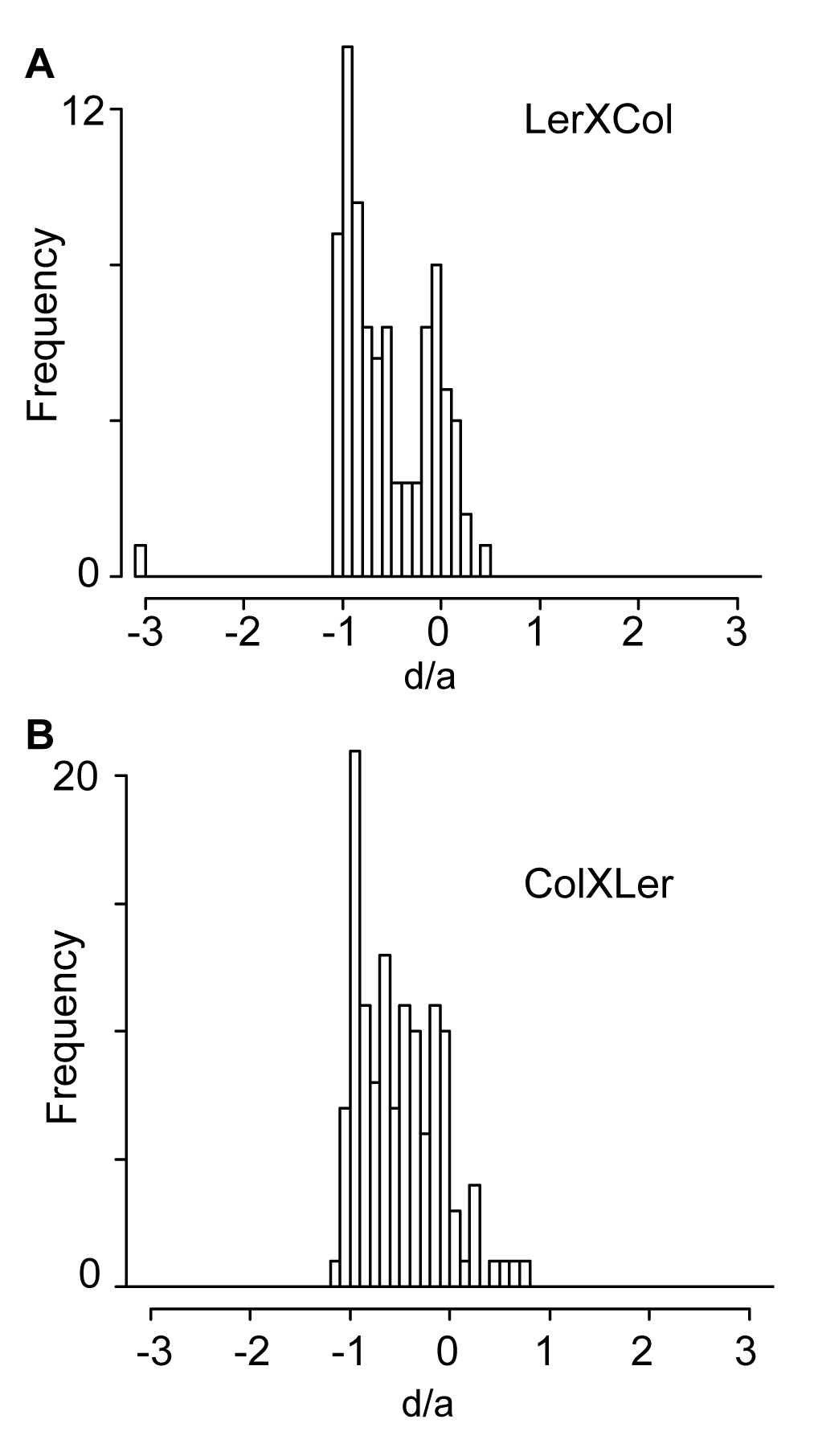

Supplement: Supplemental Figure S4 — d/a plots of set II (A) (analysis considering the hybrid with Ler as female only) and set III (B) (analysis considering the hybrid with Col as female only). d/a = 1 indicates the hybrid small RNA level is similar to the high parent while d/a = −1 indicates the hybrid small RNA level is similar to the low parent. d/a = 0 means the hybrid small RNA level is similar to the mid-parent. (TIF) [file pone.0047043.s004.tif]

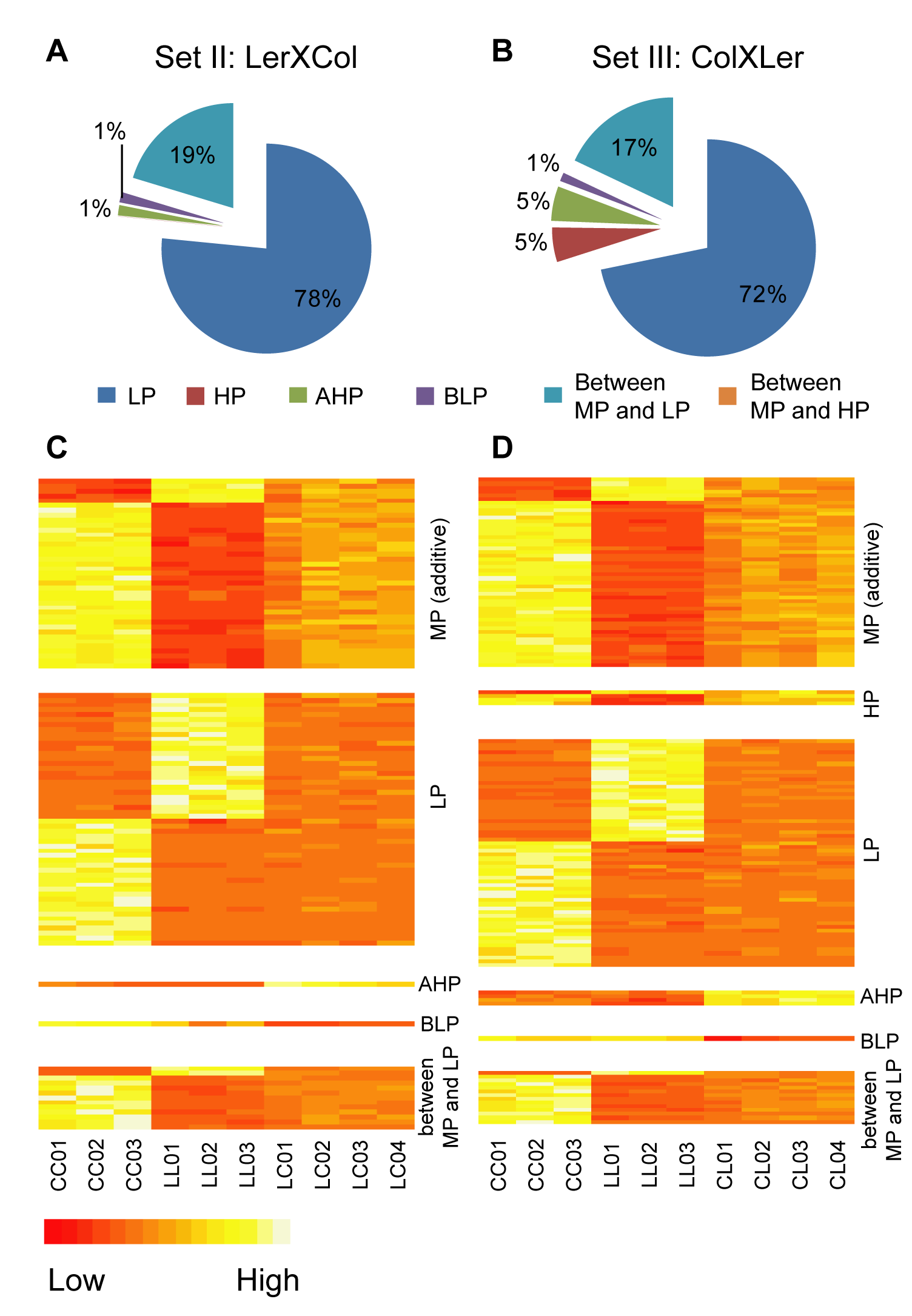

Supplement: Supplemental Figure S5 — Hybrid small RNA inheritance patterns of set II and set III. (A) Percentages of different small RNA inheritance patterns in the non-additive differentially expressed clusters (DE clusters) identified in set II (analysis considering the hybrid with Ler as female only). (B) Percentages of different patterns in the non-additive DE clusters identified in set III (analysis considering the hybrid with Col as female only). Small RNA levels of DE clusters identified in set II (C) and set III (D) were grouped by their hybrid inheritance pattern. Each row represents the normalized small RNA level (cluster count) of a DE cluster. The columns represent biological replicates. MP: mid-parent; HP: high parent; LP: low parent; AHP: above high parent; BLP: below low parent. (TIF) [file pone.0047043.s005.tif]

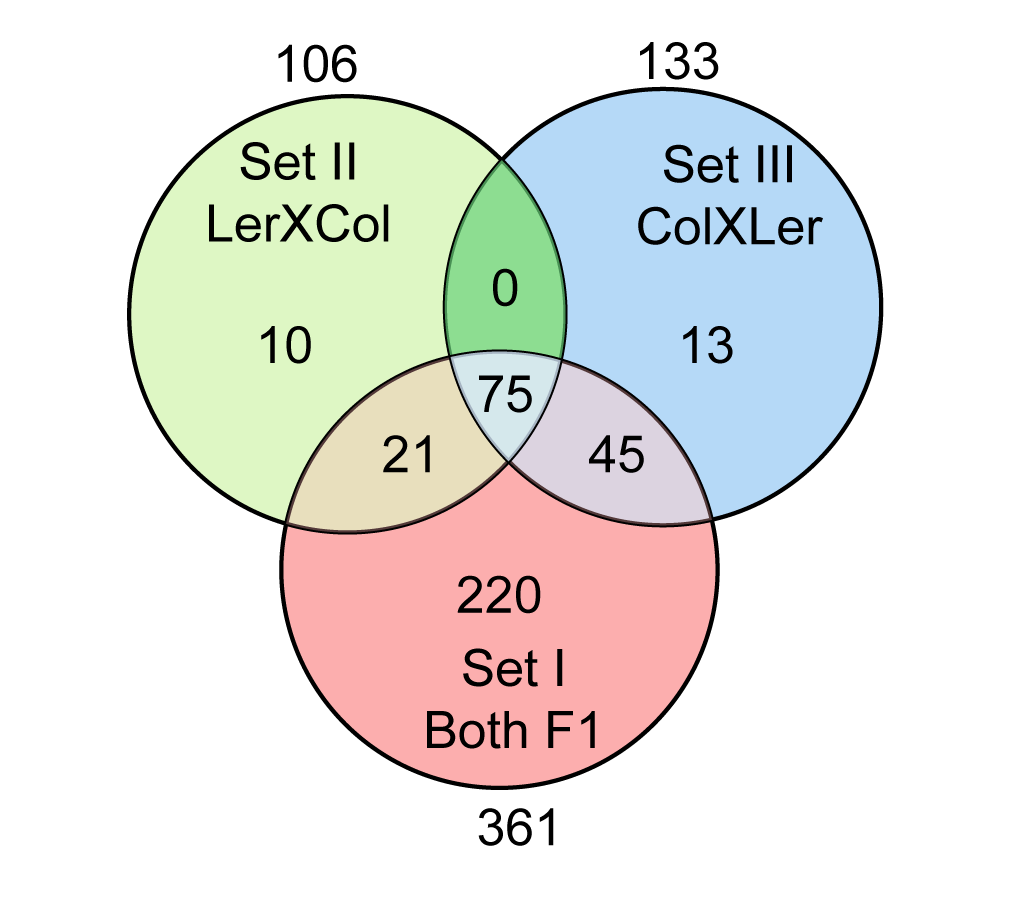

Supplement: Supplemental Figure S6 — Venn diagram showing the overlapping and unique differentially expressed clusters identified by the three analysis sets. Set I: combining both LerXCol and ColXLer as the hybrid group; Set II: only considering LerXCol (LC) as the hybrid; Set III: only considering ColXLer (CL) as the hybrid. (TIF) [file pone.0047043.s006.tif]

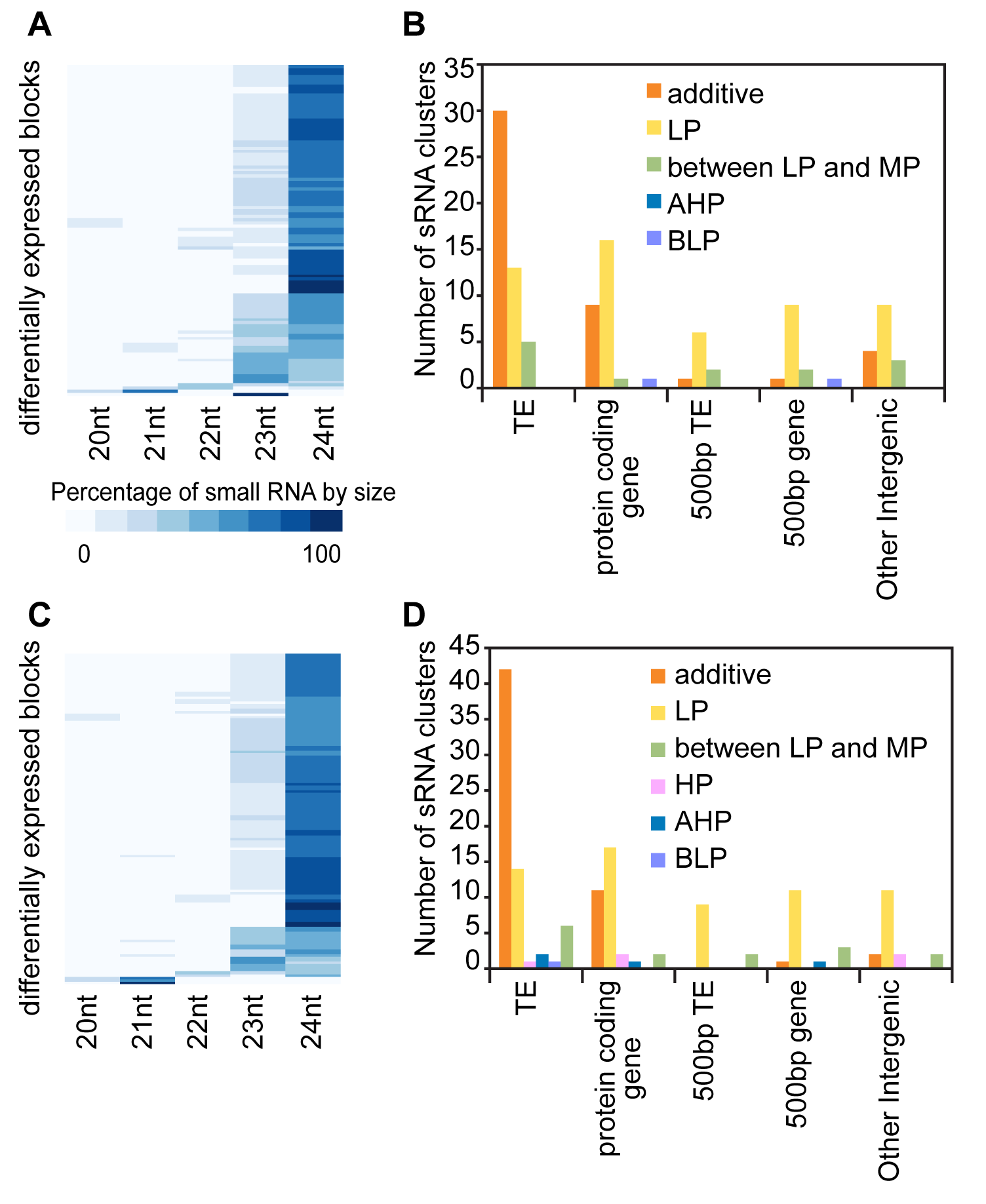

Supplement: Supplemental Figure S7 — Annotation of the differentially expressed clusters (DE clusters) in set II (analysis considering the hybrid with Ler as female only) and set III (analysis considering the hybrid with Col as female only). (A&C): Size distribution of small RNAs in the DE clusters identified in set II (A) and set III (C). (B&D): Relationship between the genomic origins and the hybrid inheritance patterns of the DE clusters identified in set II (B) and set III (D). 500 bp gene: within 500 bp upstream or downstream of a protein coding gene; 500 bp TE: within 500 bp upstream or downstream of a transposable element; LP: low parent; HP: high parent; MP: mid-parent (additive); BLP: below low parent; AHP: above high parent. (TIFF) [file pone.0047043.s007.tiff]

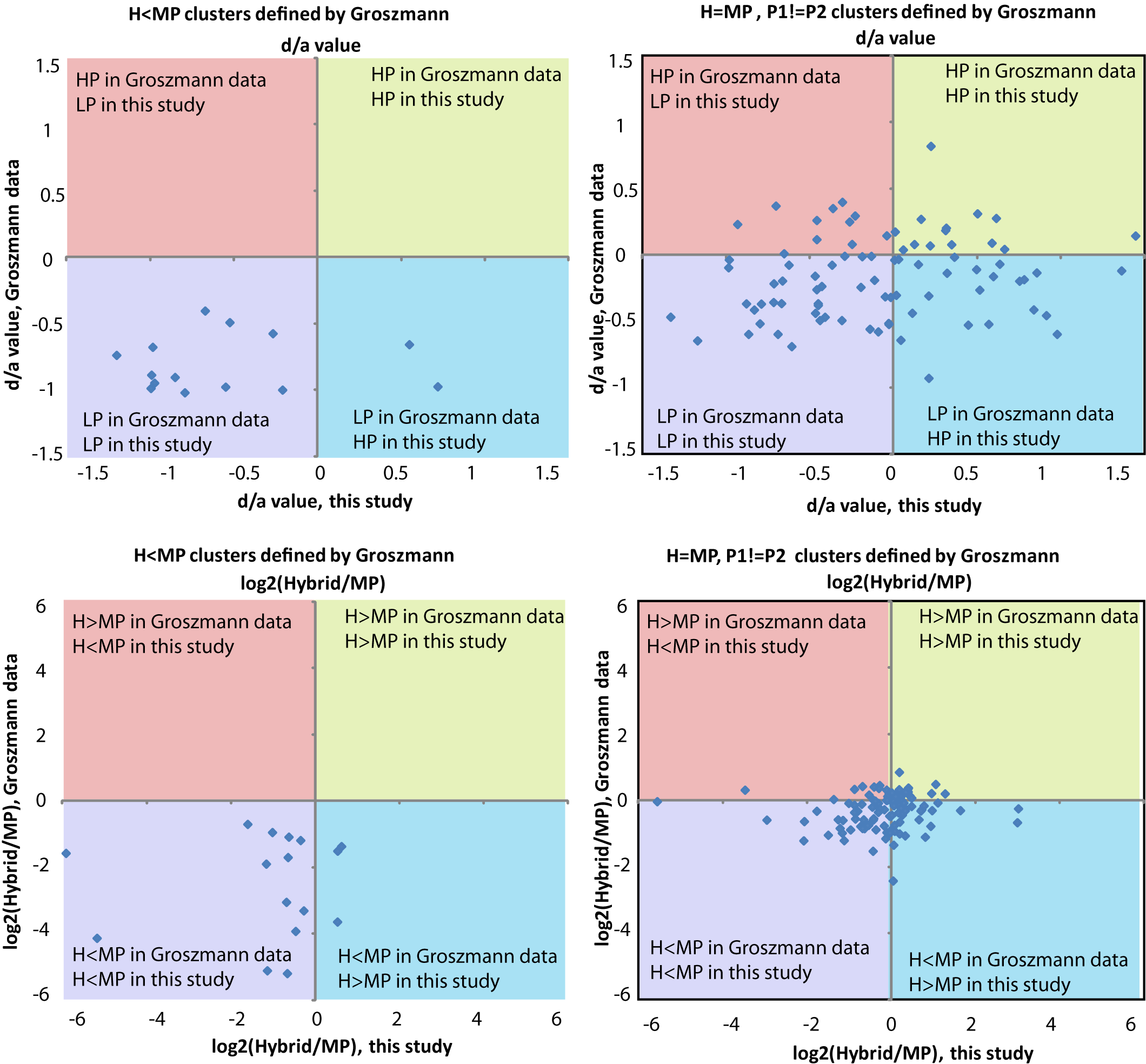

Supplement: Supplemental Figure S8 — Comparison of differentially expressed sRNA clusters identified in this replicated study compared to unreplicated dataset reported by Groszmann et al., 2011 [6] . The d/a value (A&C) and log2 of hybrid to midparent ratio (B&D) are plotted from our study (X axis) versus the Groszmann et al, 2011 study (Y axis) for both mid-parent like sRNA clusters (A&B) and lower than mid-parent sRNA clusters (C&D). H = hybrid; HP = high parent; LP = low parent; MP = midparent. (TIFF) [file pone.0047043.s008.tiff]

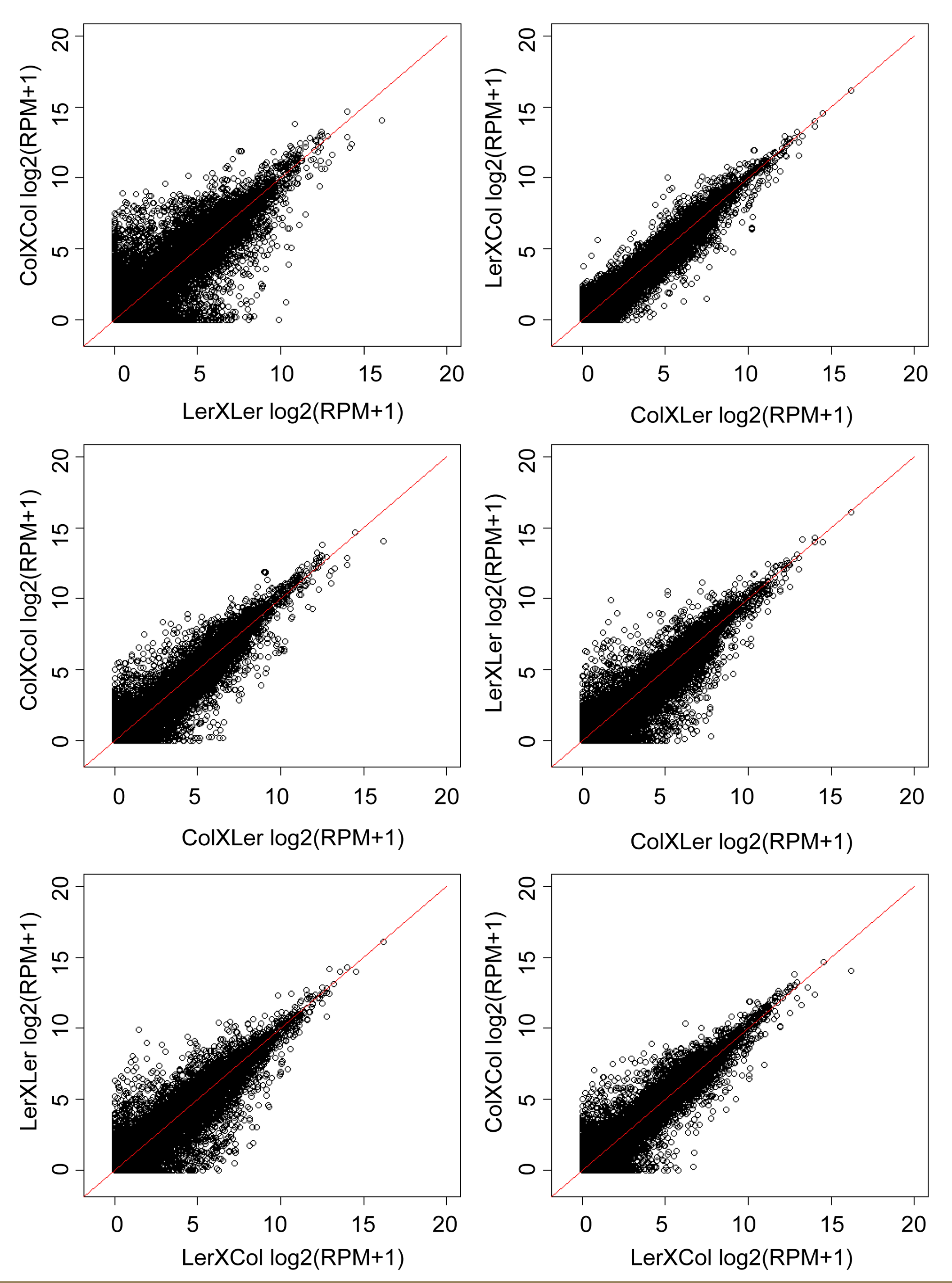

Supplement: Supplemental Figure S9 — Scatter plots showing small RNA levels of all clusters in the six binary combinations of four genotypes. In each plot, the base 2 logarithm of the mean of reads per million (RPM) value for a given cluster across biological replicates is shown. The red line represents a straight line with slope = 1. (TIF) [file pone.0047043.s009.tif]
