# Supplementary material for: The Inheritance Pattern of 24 nt siRNA Clusters in Arabidopsis Hybrids Is Influenced by Proximity to Transposable Elements
Source: PLoS One. 2012 Oct 31;7(10):e47043. doi: 10.1371/journal.pone.0047043 (PMC3485269; doi:10.1371/journal.pone.0047043)
Supplement: Table S1 — Genes identified in the differentially expressed clusters. (DOC) [file pone.0047043.s010.doc]

**Supplemental Table S1.** Genes identified in the differentially expressed blocks.

| Small RNA expression pattern | gene locus | TAIR10 gene description |
| --- | --- | --- |
| additive | AT1G14225 | unknown protein; Has 30201 Blast hits to 17322 proteins in 780 species: Archae - 12; Bacteria - 1396; Metazoa - 17338; Fungi - 3422; Plants - 5037; Viruses - 0; Other Eukaryotes - 2996 (source: NCBI BLink). |
| additive | AT1G19420 | pseudogene, CHP-rich zinc finger protein |
| additive | AT1G24388 | unknown protein; FUNCTIONS IN: molecular_function unknown; INVOLVED IN: biological_process unknown; LOCATED IN: cellular_component unknown; BEST Arabidopsis thaliana protein match is: unknown protein (TAIR:AT4G17700.1); Has 30201 Blast hits to 17322 proteins in 780 species: Archae - 12; Bacteria - 1396; Metazoa - 17338; Fungi - 3422; Plants - 5037; Viruses - 0; Other Eukaryotes - 2996 (source: NCBI BLink). |
| additive | AT1G24390 | unknown protein; Has 1 Blast hits to 1 proteins in 1 species: Archae - 0; Bacteria - 0; Metazoa - 0; Fungi - 0; Plants - 1; Viruses - 0; Other Eukaryotes - 0 (source: NCBI BLink). |
| additive | AT1G34070 | CONTAINS InterPro DOMAIN/s: Retrotransposon gag protein (InterPro:IPR005162); BEST Arabidopsis thaliana protein match is: unknown protein (TAIR:AT5G48050.1); Has 648 Blast hits to 647 proteins in 29 species: Archae - 0; Bacteria - 0; Metazoa - 16; Fungi - 25; Plants - 607; Viruses - 0; Other Eukaryotes - 0 (source: NCBI BLink). |
| additive | AT1G81020 | pseudogene, similar to Putative FH protein interacting protein FIP1, blastp match of 61% identity and 2.3e-42 P-value to GP|24756872|gb|AAN64136.1||AC121489 Putative FH protein interacting protein FIP1 {Oryza sativa (japonica cultivar-group)} |
| additive | AT2G34100 | unknown protein; Has 35333 Blast hits to 34131 proteins in 2444 species: Archae - 798; Bacteria - 22429; Metazoa - 974; Fungi - 991; Plants - 531; Viruses - 0; Other Eukaryotes - 9610 (source: NCBI BLink). |
| additive | AT3G44580 | BEST Arabidopsis thaliana protein match is: Arabidopsis retrotransposon ORF-1 protein (TAIR:AT2G14000.1); Has 12 Blast hits to 12 proteins in 2 species: Archae - 0; Bacteria - 0; Metazoa - 0; Fungi - 0; Plants - 12; Viruses - 0; Other Eukaryotes - 0 (source: NCBI BLink). |
| additive | AT4G13442 | pseudogene, similar to putative AP endonuclease/reverse transcriptase, blastp match of 37% identity and 9.1e-67 P-value to GP|21952510|gb|AAM82604.1|AF525305_2|AF525305 putative AP endonuclease/reverse transcriptase {Brassica napus} |
| additive | AT4G19270 | unknown protein; BEST Arabidopsis thaliana protein match is: unknown protein (TAIR:AT3G50250.1); Has 19 Blast hits to 14 proteins in 4 species: Archae - 0; Bacteria - 0; Metazoa - 0; Fungi - 0; Plants - 14; Viruses - 0; Other Eukaryotes - 5 (source: NCBI BLink). |
| additive | AT5G18636 | unknown protein; BEST Arabidopsis thaliana protein match is: unknown protein (TAIR:AT5G25200.1); Has 35333 Blast hits to 34131 proteins in 2444 species: Archae - 798; Bacteria - 22429; Metazoa - 974; Fungi - 991; Plants - 531; Viruses - 0; Other Eukaryotes - 9610 (source: NCBI BLink). |
| additive | AT5G28910 | unknown protein; FUNCTIONS IN: molecular_function unknown; INVOLVED IN: biological_process unknown; LOCATED IN: mitochondrion; BEST Arabidopsis thaliana protein match is: unknown protein (TAIR:AT5G28960.1); Has 35333 Blast hits to 34131 proteins in 2444 species: Archae - 798; Bacteria - 22429; Metazoa - 974; Fungi - 991; Plants - 531; Viruses - 0; Other Eukaryotes - 9610 (source: NCBI BLink). |
| additive | AT4G10201 | Pseudogene of AT3G21130; F-box family protein-related |
| additive | AT3G44510 | alpha/beta-Hydrolases superfamily protein |
| additive | AT2G17470 | Aluminium activated malate transporter family protein |
| additive | AT4G03060 | AOP2 (ALKENYL HYDROXALKYL PRODUCING 2); oxidoreductase, acting on paired donors, with incorporation or reduction of molecular oxygen, 2-oxoglutarate as one donor, and incorporation of one atom each of oxygen into both donors |
| additive | AT2G14000 | Arabidopsis retrotransposon ORF-1 protein |
| additive | AT3G44570 | Arabidopsis retrotransposon ORF-1 protein |
| additive | AT4G15320 | cellulose synthase-like B6 |
| additive | AT5G39930 | CLP1-similar protein 5 |
| additive | AT1G44020 | Cysteine/Histidine-rich C1 domain family protein |
| additive | AT5G24900 | cytochrome P450, family 714, subfamily A, polypeptide 2 |
| additive | AT1G26380 | FAD-binding Berberine family protein |
| additive | AT1G47800 | F-box and associated interaction domains-containing protein |
| additive | AT3G13680 | F-box and associated interaction domains-containing protein |
| additive | AT3G20030 | F-box and associated interaction domains-containing protein |
| additive | AT3G20690 | F-box and associated interaction domains-containing protein |
| additive | AT4G19260 | Galactose oxidase/kelch repeat superfamily protein |
| additive | AT3G29765 | General transcription factor 2-related zinc finger protein |
| additive | AT2G24720 | glutamate receptor 2.2 |
| additive | AT1G03810 | Nucleic acid-binding, OB-fold-like protein |
| additive | AT2G19360 | Protein of Unknown Function (DUF239) |
| additive | AT1G53870 | Protein of unknown function (DUF567) |
| additive | AT1G53890 | Protein of unknown function (DUF567) |
| additive | AT5G39770 | Restriction endonuclease, type II-like superfamily protein |
| additive | AT1G65350 | ubiquitin 13 |
| additive | AT4G15280 | UDP-glucosyl transferase 71B5 |
| additive | AT5G28237 | Pyridoxal-5'-phosphate-dependent enzyme family protein |
| between mid-parent and low parent | AT5G28885 | unknown protein; CONTAINS InterPro DOMAIN/s: Protein of unknown function DUF223 (InterPro:IPR003871); Has 30201 Blast hits to 17322 proteins in 780 species: Archae - 12; Bacteria - 1396; Metazoa - 17338; Fungi - 3422; Plants - 5037; Viruses - 0; Other Eukaryotes - 2996 (source: NCBI BLink). |
| between mid-parent and low parent | AT1G58808 | Unknown gene |
| between mid-parent and low parent | AT4G03050 | 2-oxoglutarate (2OG) and Fe(II)-dependent oxygenase superfamily protein |
| between mid-parent and low parent | AT2G22760 | basic helix-loop-helix (bHLH) DNA-binding superfamily protein |
| between mid-parent and low parent | AT2G21920 | F-box associated ubiquitination effector family protein |
| between mid-parent and low parent | AT5G42635 | glycine-rich protein |
| between mid-parent and low parent | AT1G59171 | Inositol-pentakisphosphate 2-kinase family protein |
| between mid-parent and low parent | AT5G36228 | nucleic acid binding;zinc ion binding |
| between mid-parent and low parent | AT3G30842 | pleiotropic drug resistance 10 |
| between mid-parent and low parent | AT4G09490 | Polynucleotidyl transferase, ribonuclease H-like superfamily protein |
| between mid-parent and low parent | AT1G43770 | RING/FYVE/PHD zinc finger superfamily protein |
| between mid-parent and low parent | AT3G09330 | Transmembrane amino acid transporter family protein |
| between mid-parent and low parent | AT1G71930 | vascular related NAC-domain protein 7 |
| Below low parent | AT3G49980 | F-box and associated interaction domains-containing protein |
| Low parent | AT1G19560 | pseudogene, putative CHP-rich zinc finger protein |
| Low parent | AT1G62863 | unknown protein; Has 30201 Blast hits to 17322 proteins in 780 species: Archae - 12; Bacteria - 1396; Metazoa - 17338; Fungi - 3422; Plants - 5037; Viruses - 0; Other Eukaryotes - 2996 (source: NCBI BLink). |
| Low parent | AT1G72080 | unknown protein; FUNCTIONS IN: molecular_function unknown; INVOLVED IN: biological_process unknown; LOCATED IN: chloroplast; Has 30201 Blast hits to 17322 proteins in 780 species: Archae - 12; Bacteria - 1396; Metazoa - 17338; Fungi - 3422; Plants - 5037; Viruses - 0; Other Eukaryotes - 2996 (source: NCBI BLink). |
| Low parent | AT2G36940 | unknown protein; Has 1 Blast hits to 1 proteins in 1 species: Archae - 0; Bacteria - 0; Metazoa - 0; Fungi - 0; Plants - 1; Viruses - 0; Other Eukaryotes - 0 (source: NCBI BLink). |
| Low parent | AT3G25130 | unknown protein; FUNCTIONS IN: molecular_function unknown; INVOLVED IN: biological_process unknown; LOCATED IN: plasma membrane; EXPRESSED IN: 17 plant structures; EXPRESSED DURING: 9 growth stages; Has 3885 Blast hits to 2658 proteins in 280 species: Archae - 12; Bacteria - 208; Metazoa - 970; Fungi - 222; Plants - 148; Viruses - 11; Other Eukaryotes - 2314 (source: NCBI BLink). |
| Low parent | AT3G48650 | pseudogene, At14a-related protein, similar to At14a (GI:11994571 and GI:11994573) (Arabidopsis thaliana) |
| Low parent | AT4G10080 | unknown protein; BEST Arabidopsis thaliana protein match is: unknown protein (TAIR:AT4G13530.1); Has 120 Blast hits to 114 proteins in 21 species: Archae - 2; Bacteria - 4; Metazoa - 0; Fungi - 12; Plants - 100; Viruses - 0; Other Eukaryotes - 2 (source: NCBI BLink). |
| Low parent | AT2G03291 | Pseudogene of AT2G22340; unknown protein |
| Low parent | AT5G60440 | AGAMOUS-like 62 |
| Low parent | AT1G56630 | alpha/beta-Hydrolases superfamily protein |
| Low parent | AT3G02410 | alpha/beta-Hydrolases superfamily protein |
| Low parent | AT4G17970 | aluminum-activated, malate transporter 12 |
| Low parent | AT1g34050 | Ankyrin repeat family protein |
| Low parent | AT1G49250 | ATP-dependent DNA ligase |
| Low parent | AT5G47130 | Bax inhibitor-1 family protein |
| Low parent | AT2G44470 | beta glucosidase 29 |
| Low parent | AT4G32105 | Beta-1,3-N-Acetylglucosaminyltransferase family protein |
| Low parent | AT1G67980 | caffeoyl-CoA 3-O-methyltransferase |
| Low parent | AT4G13992 | Cysteine/Histidine-rich C1 domain family protein |
| Low parent | AT1G51150 | DegP protease 6 |
| Low parent | AT1G56520 | Disease resistance protein (TIR-NBS-LRR class) family |
| Low parent | AT4G16950 | Disease resistance protein (TIR-NBS-LRR class) family |
| Low parent | AT1G61320 | FBD / Leucine Rich Repeat domains containing protein |
| Low parent | AT2G38590 | F-box and associated interaction domains-containing protein |
| Low parent | AT3G21170 | F-box and associated interaction domains-containing protein |
| Low parent | AT5G66380 | folate transporter 1 |
| Low parent | AT1G19470 | Galactose oxidase/kelch repeat superfamily protein |
| Low parent | AT3G60140 | Glycosyl hydrolase superfamily protein |
| Low parent | AT1G63950 | Heavy metal transport/detoxification superfamily protein |
| Low parent | AT3G46340 | Leucine-rich repeat protein kinase family protein |
| Low parent | AT3G04290 | Li-tolerant lipase 1 |
| Low parent | At4G29300 | low-molecular-weight cysteine-rich 27 |
| Low parent | AT4G15760 | monooxygenase 1 |
| Low parent | AT4G12720 | MutT/nudix family protein |
| Low parent | AT2G19800 | myo-inositol oxygenase 2 |
| Low parent | AT5G06510 | nuclear factor Y, subunit A10 |
| Low parent | AT4G32375 | Pectin lyase-like superfamily protein |
| Low parent | AT3G61530 | Phosphoenolpyruvate carboxylase family protein |
| Low parent | AT4G39810 | Polynucleotidyl transferase, ribonuclease H-like superfamily protein |
| Low parent | AT3G46160 | Protein kinase superfamily protein |
| Low parent | AT4G21366 | Protein kinase superfamily protein |
| Low parent | AT2G43470 | Protein of unknown function (DUF3755) |
| Low parent | AT3G49070 | Protein of unknown function (DUF677) |
| Low parent | AT3G18260 | Reticulon family protein |
| Low parent | AT3G04180 | RmlC-like cupins superfamily protein |
| Low parent | AT4G21370 | S-locus protein kinase, putative |
| Low parent | AT1G61120 | terpene synthase 04 |
| Low parent | AT4G13280 | terpenoid synthase 12 |
| Low parent | AT4G13300 | terpenoid synthase 13 |
| Low parent | AT4G00230 | xylem serine peptidase 1 |
| Low parent | AT4G28850 | xyloglucan endotransglucosylase/hydrolase 26 |
| Low parent | AT2G36870 | xyloglucan endotransglucosylase/hydrolase 32 |
| Low parent | AT2G01973 | pseudogene of OTU-like cysteine protease family protein |
