# Supplementary material for: The Inheritance Pattern of 24 nt siRNA Clusters in Arabidopsis Hybrids Is Influenced by Proximity to Transposable Elements
Source: PLoS One. 2012 Oct 31;7(10):e47043. doi: 10.1371/journal.pone.0047043 (PMC3485269; doi:10.1371/journal.pone.0047043)
Supplement: Table S2 — GO terms identified among the genes associated with down-regulated small RNAs. Plot X and plot Y are the coordinators of the GO terms in the semantic space, generated by REVIGO. The p-value was calculated by hypergeometric distribution with the Arabidopsis whole gene set as background. (DOC) [file pone.0047043.s011.doc]

**Supplemental Table S2**. GO terms identified among the genes associated with down-regulated small RNAs. Plot X and plot Y are the coordinators of the GO terms in the semantic space, generated by REVIGO. The p-value was calculated by hypergeometric distribution with the Arabidopsis whole gene set as background.

| GO term ID | GO term description | plot_X | plot_Y | log10 p-value |
| --- | --- | --- | --- | --- |
| GO:0045338 | farnesyl diphosphate metabolic process | 4.699 | 0.358 | -4.8813 |
| GO:0016106 | sesquiterpenoid biosynthetic process | 5.156 | 2.496 | -4.5812 |
| GO:0006073 | cellular glucan metabolic process | 3.115 | 6.26 | -2.9731 |
| GO:0009611 | response to wounding | -5.434 | 4.067 | -2.6813 |
| GO:0015884 | folic acid transport | -2.431 | -5.377 | -2.6751 |
| GO:0016102 | diterpenoid biosynthetic process | 5.24 | 2.345 | -2.6751 |
| GO:0005975 | carbohydrate metabolic process | 4.284 | 5.599 | -2.2142 |
| GO:0015940 | pantothenate biosynthetic process | 4.883 | 1.728 | -2.0758 |
| GO:0000304 | response to singlet oxygen | -5.586 | 2.64 | -1.9015 |
| GO:0006916 | anti-apoptosis | -0.323 | -0.254 | -1.9015 |
| GO:0006725 | cellular aromatic compound metabolic process | 1.791 | 5.054 | -1.7784 |
| GO:0010089 | xylem development | 2.22 | -4.901 | -1.5408 |
| GO:0009870 | defense response signaling pathway, resistance gene-dependent | -4.371 | 4.487 | -1.5117 |
| GO:0009960 | endosperm development | 2.592 | -5.065 | -1.4591 |
| GO:0009741 | response to brassinosteroid stimulus | -6.326 | 1.959 | -1.4352 |
| GO:0006310 | DNA recombination | 3.017 | 7.209 | -1.371 |
| GO:0010193 | response to ozone | -6.077 | 1.421 | -1.3333 |
| GO:0006629 | lipid metabolic process | 3.32 | 4.908 | -1.2709 |
| GO:0009626 | plant-type hypersensitive response | -4.678 | 4.687 | -1.1735 |
| GO:0009809 | lignin biosynthetic process | 4.725 | 2.187 | -1.1505 |
| GO:0006486 | protein glycosylation | 4.543 | 4.954 | -1.1395 |
| GO:0009817 | defense response to fungus, incompatible interaction | -5.455 | 4.753 | -1.0796 |
| GO:0009744 | response to sucrose stimulus | -5.847 | 1.885 | -1.0531 |
| GO:0006260 | DNA replication | 3.662 | 5.923 | -1.0364 |
| GO:0007568 | aging | 2.604 | -4.809 | -1.0205 |
| GO:0006865 | amino acid transport | -2.471 | -5.415 | -0.9979 |
| GO:0008150 | biological_process | 1.267 | -3.292 | -0.9877 |
| GO:0009735 | response to cytokinin stimulus | -6.045 | 2.264 | -0.9767 |
| GO:0006855 | drug transmembrane transport | -4.964 | 0.617 | -0.9379 |
| GO:0009617 | response to bacterium | -5.313 | 3.538 | -0.8622 |
| GO:0030001 | metal ion transport | -2.739 | -4.902 | -0.8346 |
| GO:0035556 | intracellular signal transduction | -3.946 | 4.779 | -0.7937 |
| GO:0006952 | defense response | -6.018 | 4.027 | -0.7871 |
| GO:0006281 | DNA repair | -2.038 | 4.745 | -0.7861 |
| GO:0006508 | proteolysis | 4.395 | 6.19 | -0.7779 |
| GO:0006468 | protein phosphorylation | 2.975 | 6.255 | -0.7564 |
| GO:0009753 | response to jasmonic acid stimulus | -6.513 | 2.524 | -0.6931 |
| GO:0009416 | response to light stimulus | -6.517 | 3.679 | -0.6829 |
| GO:0007275 | multicellular organismal development | 2.393 | -4.962 | -0.636 |
| GO:0042742 | defense response to bacterium | -5.768 | 4.339 | -0.634 |
| GO:0006979 | response to oxidative stress | -5.869 | 3.322 | -0.5334 |
| GO:0009737 | response to abscisic acid stimulus | -6.222 | 2.802 | -0.5241 |
| GO:0006355 | regulation of transcription, DNA-dependent | 2.478 | 5.136 | -0.4595 |
| GO:0008152 | metabolic process | 0.567 | -2.615 | -0.4568 |
| GO:0009651 | response to salt stress | -6.191 | 4.187 | -0.4547 |
| GO:0006810 | transport | -2.659 | -5.024 | -0.44 |
| GO:0006499 | N-terminal protein myristoylation | 4.726 | 4.152 | -0.4321 |
